# Multi‐Omics Analysis and Real‐World Data Validation of Serine Metabolism‐Related Genes in Colorectal Cancer

**DOI:** 10.1111/jcmm.70721

**Published:** 2025-07-14

**Authors:** Anqi Li, Qihui Wu, Yuanyuan Xu, Yijin Gu, Xuan Wang, Jiaxin Liu, Yan Wang, Jialing Xie, Xiaodan Fu, Yimin Li

**Affiliations:** ^1^ Department of Pathology Ruijin Hospital, Shanghai Jiaotong University School of Medicine Shanghai China; ^2^ Department of Gynecology Xiangya Hospital, Central South University Changsha China; ^3^ National Clinical Research Center for Geriatric Disorders Xiangya Hospital Changsha China; ^4^ Department of Pathology School of Basic Medical Sciences, Central South University Changsha China; ^5^ Department of Pathology Xiangya Hospital, Central South University Changsha China

**Keywords:** colorectal cancer, Pan‐cancer, prognosis, serine metabolism, tertiary lymphoid structures, tumour microenvironment

## Abstract

Serine metabolism plays a pivotal role in cancer progression by supporting essential biosynthetic pathways and energy production. Exploring the intricacies of serine metabolism in cancer may uncover novel therapeutic opportunities. This study presents a comprehensive pan‐cancer analysis of serine metabolism‐related genes (SMGs), with a particular emphasis on colorectal cancer (CRC), to elucidate their expression patterns, genetic alterations and clinical significance. We performed a pan‐cancer analysis of SMGs using integrating transcriptomic, genomic and epigenetic data from TCGA and GTEx databases. For CRC, we performed in‐depth analyses comparing expression patterns between tumour and normal tissues, examining prognostic significance and exploring associations with the tumour microenvironment (TME). The distribution patterns of SMGs within the TME were further investigated using single‐cell RNA sequencing and immunohistochemistry. Key SMGs, including PHGDH, SLC1A5 and SLC38A2, were validated in two independent real‐world cohorts of CRC. Pan‐cancer analysis revealed that SMGs are differentially expressed across tumour types, with their dysregulation associated with copy number alterations and epigenetic modifications. In CRC, aberrant SMG expression is significantly associated with clinical outcomes, key signalling pathways and the TME. Notably, PHGDH was consistently upregulated in CRC and associated with poor prognosis, while SLC1A5 emerged as a potential biomarker for liver metastasis. This study underscores the importance of SMGs, particularly PHGDH, SLC1A5 and SLC38A2, in CRC progression and prognosis. Our findings offer valuable insights into SMGs as a potential therapeutic target and provide a foundation for developing personalised metabolic interventions in CRC.

**Abbreviations**
3‐PG: 3‐phosphoglycerate
3‐PHP: 3‐phosphohydroxypyruvate
3‐PS: 3‐phosphoserine
ACC: adrenocortical carcinoma
BLCA: bladder urothelial carcinoma
BRCA: breast invasive carcinoma
CESC: cervical squamous cell carcinoma and endocervical adenocarcinoma
CHOL: cholangiocarcinoma
CMS: consensus molecular subtypes
CNV: copy number variation
COAD: colon adenocarcinoma
CRC: colorectal cancer
DCs: dendritic cells
Depmap: The Cancer Dependency Map
DLBC: lymphoid neoplasm diffuse large B‐cell lymphoma
EMT: epithelial‐mesenchymal transition
ESCA: oesophageal carcinoma
GCs: germinal centres
GEO: gene expression omnibus
GBM: glioblastoma multiforme
GSEA: gene set enrichment analysis
GTEx: genotype‐tissue expression project
HNSC: head and neck squamous cell carcinoma
iTLS: immature TLS
KICH: kidney renal chromophobe
KIRC: kidney renal clear cell carcinoma
KIRP: kidney renal papillary cell carcinoma
HPA: Human Protein Atlas
IHC: immunohistochemistry
LAML: acute myeloid leukaemia
LGG: lower grade glioma
LIHC: liver hepatocellular carcinoma
LUAD: lung adenocarcinoma
LUSC: lung squamous cell carcinoma
m1A: N1‐methyladenosine
m5C: 5‐methylcytosine
m6A: N6‐methyladenosine
MSI: microsatellite instability
MSS: microsatellite stable
mTLS: mature TLS
NES: normalised enrichment score
MESO: mesothelioma
OV: ovarian serous cystadenocarcinoma
PAAD: pancreatic adenocarcinoma
PCPG: pheochromocytoma and paraganglioma
PFL: primary follicles
PHGDH: phosphoglycerate dehydrogenase
pMMR: proficient mismatch repair
PRAD: prostate adenocarcinoma
PSAT1: phosphoserine aminotransferase 1
PSPH: phosphoserine phosphatase
READ: rectal adenocarcinoma
SARC: sarcoma
SFL: secondary follicles
SSP: serine synthesis pathway
scRNA‐seq: single‐cell RNA sequencing
SKCM: skin cutaneous melanoma
SLC1A5: solute carrier family 1 member 5
SLC38A2: solute carrier family 38 member 2
SMGs: serine metabolism‐related genes
STAD: stomach adenocarcinoma
TCGA: The Cancer Genome Atlas
TFH: T follicular helper
TGCT: testicular germ cell tumours
THCA: thyroid carcinoma
THYM: thymoma
TISCH2: tumour immune single‐cell hub 2
TLSs: tertiary lymphoid structures
TME: tumour immune microenvironment
TPM: transcripts per kilobase million
UCEC: uterine corpus endometrial carcinoma
UCS: uterine carcinosarcoma
UVM: uveal melanoma

## 1 Introduction

Cancer cells undergo profound metabolic reprogramming to sustain their survival and rapid proliferation, enabling the production of energy and macromolecules essential for growth [1]. Among the key metabolites, serine serves as a crucial exogenous precursor for proteins, lipids and nucleotides, driving proliferation in diverse tumour types including colorectal cancer (CRC) [2, 3, 4]. As a nonessential amino acid, serine can be acquired exogenously through dietary intake or synthesised endogenously via the serine synthesis pathway (SSP) [5]. The potential of targeting serine metabolism—either through dietary restriction or inhibition of its endogenous synthesis—has garnered increasing interest in cancer research. Elucidating the regulatory mechanisms governing serine metabolism in cancer is therefore essential for advancing both fundamental research and precision oncology approaches [6].

The SSP is mediated by three key enzymes: phosphoglycerate dehydrogenase (PHGDH), phosphoserine aminotransferase 1 (PSAT1) and phosphoserine phosphatase (PSPH). This pathway utilises the glycolytic or gluconeogenic intermediate 3‐phosphoglycerate (3‐PG) as a substrate [6, 7]. PHGDH catalyses the NAD + ‐dependent oxidation of 3‐PG to 3‐phosphohydroxypyruvate (3‐PHP), which is then converted to 3‐phosphoserine (3‐PS) by PSAT1 via a glutamate‐dependent transamination reaction. Finally, PSPH catalyses the hydrolysis of 3‐PS to produce serine [8]. Substantial evidence indicates that upregulation of these enzymes in multiple malignancies, including CRC, breast cancer, melanoma and nonsmall cell lung cancer, plays critical roles in tumour growth and drug resistance [2, 5, 9, 10, 11]. Therefore, therapeutic targeting of the key enzymes of the SSP, particularly PHGDH inhibition, has gained significant interest [5]. However, inhibiting PHGDH may trigger compensatory mechanisms, such as increased extracellular serine uptake from the tumour microenvironment (TME) [6, 12]. Notably, dietary restriction of serine and glycine suppresses tumour growth and extends survival in several mouse cancer models [5, 13]. Thus, dual blockade of exogenous serine uptake and endogenous biosynthesis may represent a more effective anticancer strategy [3].

Amino acid transporters are membrane‐bound proteins regulating cellular and organellar amino acid flux [14]. While the exact transporters involved in serine uptake within tumours remain incompletely defined [15], members of the solute carrier (SLC) superfamily, such as SLC1A4 (ASCT1) [16], SLC1A5 (ASCT2) [15, 17, 18], SLC7A10 [19], SLC38A2 [20], SLC38A4 [14] and SLC38A5 [14], have been implicated. Among these, SLC1A4 and SLC1A5 are key transporters of neutral amino acids and are frequently upregulated in tumours, correlating with poor prognosis [15, 21, 22]. Notably, under conditions of serine deprivation, loss of SLC1A5 (ASCT2) has been shown to inhibit cancer cell growth [15]. Therefore, understanding the sources and regulatory mechanisms of serine in specific tumours is critical for developing therapeutic treatment strategies.

Beyond fulfilling the intrinsic metabolic needs of tumour cells, serine metabolism also plays a pivotal role in shaping the TME [23, 24]. Serine availability influences both innate and adaptive immune responses by modulating immune cell proliferation, differentiation and effector function [24, 25, 26, 27]. Notably, tertiary lymphoid structures (TLSs)—ectopic lymphoid aggregates formed in nonlymphoid tissues under chronic inflammatory conditions, including tumours—have gained recognition as key immune niches within the TME [28, 29]. Their presence has been correlated with favourable prognosis and improved response to immunotherapy in several cancers, including CRC [30, 31, 32, 33]. Considering that serine metabolism regulates multiple immune cell types, elucidating its role in the development, maintenance and function of TLSs may reveal new opportunities to enhance the synergy between metabolic modulation and immunotherapeutic strategies.

In this study, we conducted a comprehensive pan‐cancer analysis of serine metabolism‐related genes (SMGs) to elucidate their expression and regulatory patterns across various tumours. With a particular focus on CRC, we delved deeper into the expression profiles of these genes and examined their associations with clinical prognosis, biological function and the TME, and validated key genes (PHGDH, SLC1A5, SLC38A2) in real‐world CRC cohorts. Through integrated multi‐omics analysis and validation, our study enhances the overall understanding of the role of SMGs in cancer progression and offers new insights for developing targeted therapies against serine metabolism in cancer.

## 2 Materials and Methods

### 2.1 Data Acquisition and Processing

To provide a clear overview of the study design and analytical workflow, we summarised the entire process in a schematic diagram (Figure S1). RNA sequencing (TPM value), somatic mutation profiles, copy number variation (CNV), DNA methylation and clinical information for 33 cancer types from The Cancer Genome Atlas (TCGA), as well as normal tissue data from 31 tissue types in the Genotype‐Tissue Expression (GTEx) project, were retrieved from the UCSC Xena database (https://xenabrowser.net/datapages/). CRISPR‐Cas9 gene dependency scores for CRC cell lines were sourced from The Cancer Dependency Map (DepMap) database (https://depmap.org/portal/). Additionally, 12 microarray datasets (GSE110224, GSE21510, GSE32323, GSE39582, GSE9348, GSE17536, GSE17537, GSE29621, GSE38832, GSE72970, GSE41258, GSE49355) were obtained from the Gene Expression Omnibus (GEO) to validate the expression and prognostic relevance of SMGs in CRC. Batch effects across these datasets were corrected using the ‘ComBat’ algorithm, minimising nonbiological technical variations [34]. After batch effect correction, we compiled the ‘meta.data’ cohort, consisting of samples from the TCGA‐COAD, TCGA‐READ and six GEO datasets (GSE17536, GSE17537, GSE29621, GSE38832, GSE39582, GSE72970), all of which provided complete overall survival (OS) information. To investigate the potential roles of SMGs in therapeutic responses, we collected six GEO datasets from patients who received fluorouracil‐based adjuvant chemotherapy (ACT) (FOLFOX or FOLFIRI) alone (GSE19860, GSE28702, GSE45404, GSE62080, GSE69657, GSE72970), as well as three datasets involving combined treatment with fluorouracil‐based ACT and bevacizumab (GSE19860, GSE19862, GSE72970). Protein expression data of SMGs were obtained from the Human Protein Atlas (HPA: https://www.proteinatlas.org/). Lists of serine transmembrane transporters were sourced from the SLC Tables (http://slc.bioparadigms.org/) [14]. Lists of genes associated with methylation regulators, including N1‐methyladenosine (m1A), 5‐methylcytosine (m5C) and N6‐methyladenosine (m6A), were obtained from SangerBox3.0 (http://sangerbox.com/).

### 2.2 Human Tissue Procurement

Two independent sets of human tissue samples were utilised in this study. Cohort 1 comprised 11 matched CRC samples, including normal colorectal tissue, primary tumour tissue and corresponding liver metastases, obtained from Ruijin Hospital, Shanghai Jiao Tong University School of Medicine. Cohort 2 included tissue microarrays from Xiangya Hospital, Central South University, comprising 41 normal colorectal tissues and 113 CRC tissues. Ethical approval for the use of human specimens was granted by the institutional review boards of both participating institutions. All patients provided written informed consent, and no patients received preoperative chemotherapy or radiotherapy.

### 2.3 Prognostic Analysis

Two approaches were employed for survival analysis: univariate Cox regression analysis using the ‘coxph’ function from the ‘survival’ R package and the Kaplan–Meier method implemented with the ‘survival’ and ‘survminer’ R packages. The analysis was conducted with a minimum sample size that exceeded 20% of each group to ensure statistical robustness. The results were visualised as a heatmap using the ‘ComplexHeatmap’ R package.

### 2.4 Tumour Microenvironment Analysis

The TME has been recognised as a critical factor influencing cancer progression and therapeutic response [35, 36, 37]. To assess immune, stromal, and tumour purity scores, the ESTIMATE algorithm was utilised [38]. Pathway scores for TME‐related pathways were calculated using the IOBP algorithm [38]. Immune cell infiltration analysis was conducted using the MCP‐counter algorithm, which estimates the abundance of specific immune cell populations within the TME [39, 40].

### 2.5 Single‐Cell Analysis

Recent advances in single‐cell RNA sequencing (scRNA‐seq) have paved the way for a new era of precision oncology [41]. Therefore, we analysed the expression of SMGs across different cell types at the single‐cell level. The CRC scRNA‐seq data were obtained from the tumour immune single‐cell hub 2 (TISCH2) (http://tisch.comp‐genomics.org/documentation/), a specialised database for TME exploration across various cancer types [42]. All datasets in TISCH2 were processed using a standardised bioinformatics pipeline based on MAESTRO v1.1.0, which incorporates key steps including quality control, batch effect removal, cell clustering, differential expression analysis, cell‐type annotation and malignant cell classification [42, 43]. In this study, we directly downloaded the processed gene expression data of SMGs in CRC from TISCH2 and visualised the data using the ‘ComplexHeatmap’ R package. A UMAP plot was generated to illustrate the expression distribution of these genes in different cell types in the CRC_GSE166555 dataset.

### 2.6 Functional Enrichment Analysis

Gene set enrichment analysis (GSEA) was performed using the ‘clusterProfiler’ R package [44]. For each cancer type, samples were ranked based on gene expression levels, and the top 30% and bottom 30% of samples were classified as high‐expression and low‐expression groups respectively. Differential expression analysis was then conducted using the ‘limma’ R package, with genes meeting an adjusted *p* value threshold of < 0.05 considered significant. The hallmark gene set ‘h.all.v7.5.1.symbols’ from the MSigDB database (https://www.gsea‐msigdb.org/gsea/msigdb/) served as the reference for enrichment analysis. Enrichment results were evaluated by normalised enrichment scores (NES) and false discovery rates (FDR).

### 2.7 Immunohistochemistry

Immunohistochemistry (IHC) was performed according to established protocols [45]. Four‐micrometre‐thick tissue sections were first deparaffinised and hydrated. Antigen retrieval was conducted using microwave heating in sodium citrate buffer. Endogenous peroxidase activity was blocked with a 3% hydrogen peroxide solution for 10 min. To reduce nonspecific binding, sections were incubated with 5% bovine serum albumin (BSA). Primary antibodies against PHGDH (1:300, A22129, Abclonal, China), SLC1A5 (1:400, A23156, Abclonal, China), SLC38A2 (1:300, BMP081, MBL, Japan) and Ki67 (1:250, 100,130‐MM19, SinoBiological, China) were applied and incubated overnight at 4°C. Subsequently, sections were incubated with a polymer labelled with anti‐mouse/rabbit antibodies for 20 min. Target proteins were visualised using 0.01% DAB staining solution, and nuclei were counterstained with haematoxylin. The stained sections were evaluated by two independent pathologists blinded to clinical data. Staining intensity was scored as follows: 0 (no staining), 1 (weak staining), 2 (moderate staining) and 3 (strong staining). The IHC staining score (IHCscore) was calculated by multiplying the staining intensity by the percentage of positive tumour cells.

### 2.8 Statistical Analysis

Statistical analyses were performed using R software (version 4.2.2) and relevant online tools. For comparisons between two groups, Student's t‐test or Wilcoxon test was used, while one‐way ANOVA or Kruskal–Wallis test was employed for multiple group comparisons. Correlations between continuous variables were determined using Spearman's correlation test. Statistical significance was defined as *p* < 0.05, with significance levels indicated as follows: *p < 0.05, **p < 0.01, ***p < 0.001 and ****p < 0.0001.

## 3 Results

### 3.1 Pan‐Cancer Analysis of SMGs Expression

**FIGURE 1:**
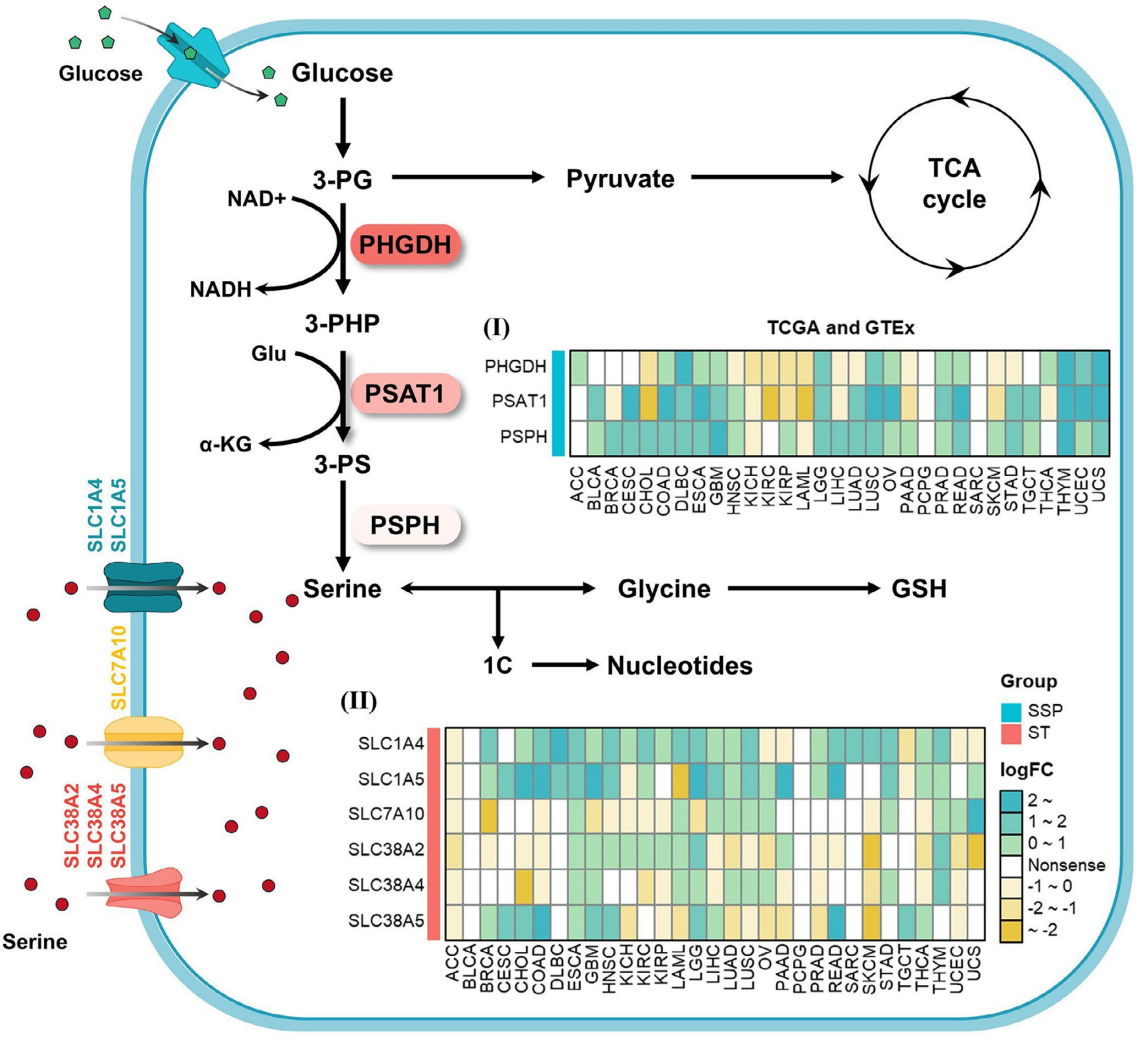
Expression of key enzymes in SMGs across pan‐cancer types. Schematic representation of the serine metabolism pathways, showing the serine synthesis pathway (SSP) (top left) and serine transmembrane transport (ST, bottom left). The heatmap on the right displays the expression levels of key enzymes in the SSP (Panel I) and ST (Panel II) across various tumour and normal tissues, based on data from the TCGA and GTEx databases. Colours represent fold changes, and ‘nonsense’ indicates *p* values > 0.05.

As a nonessential amino acid, serine can be synthesised via the SSP or imported into cells through specific serine transporters (Figure 1). To thoroughly investigate the expression patterns of SMGs, we analysed their transcriptional profiles across various tumours using integrated TCGA pan‐cancer and GTEx databases. While prior studies established upregulation of PHGDH, PSAT1 and PSPH in certain cancers [3], our pan‐cancer analyses reveal distinct expression of these genes in different tumour types: elevated expression was observed in COAD, DLBC, ESCA, GBM, LGG, LUSC, OV, PRAD, READ, THYM, UCEC and UCS, while suppressed expression was observed in KICH, KIRC, KIRP and LAML (Figure 1 (I)). In addition to the SSP, members of the SLC transporter family contribute to maintaining serine homeostasis through extracellular uptake [14, 15, 46]. Notably, SLC1A4 and SLC1A5 demonstrate consistent upregulation, particularly in CHOL, COAD and GBM (Figure 1 (II)), whereas SLC7A10, SLC38A2, SLC38A4 and SLC38A5 exhibit tumour‐type‐specific expression patterns (Figure 1 (II)). Further differential expression analyses based on both unpaired and paired TCGA pan‐cancer samples revealed dysregulation of SMGs across various tumours (Figure S2A–C). Data from the human Cancer Cell Line Encyclopedia (CCLE) demonstrated differential expression of these genes across cell lines, with high expression of PHGDH, PSAT1, SLC1A5 and SLC38A2, and low expression of SLC38A4 and SLC7A10 (Figure S3). Critically, PHGDH, PSAT1, PSPH, SLC1A4, SLC1A5 and SLC38A2 show robust co‐expression patterns in both TCGA tumour samples and CCLE cancer cell lines (Figure S4A–B), suggesting coregulated functionality.

Pan‐cancer mutational profiling of SMGs revealed low mutation frequencies in most genes, except for a higher mutation frequency of SLC38A4 in SKCM (Figure S5A). Additionally, CNV appeared to be associated with the expression of these genes in several tumours (Figure S5B). Notably, PSPH mRNA expression was positively correlated with CNV in multiple cancers, including GBM (Figure S5B). We further explored the relationship between the expression of SMGs and epigenetic modifications in pan‐cancer. The results showed that the expression of PSAT1 and SLC7A10 was inversely correlated with their DNA methylation levels (Figure S5C). Conversely, the expression levels of PSPH and SLC38A2 were positively associated with the activity of RNA‐modifying enzymes involved in modifications such as m1A, m5C and m6A (Figure S6A). These results suggest that both CNV and epigenetic modifications may contribute to the dysregulated expression of SMGs in tumours.

### 3.2 Expression Patterns of SMGs in CRC

**FIGURE 2:**
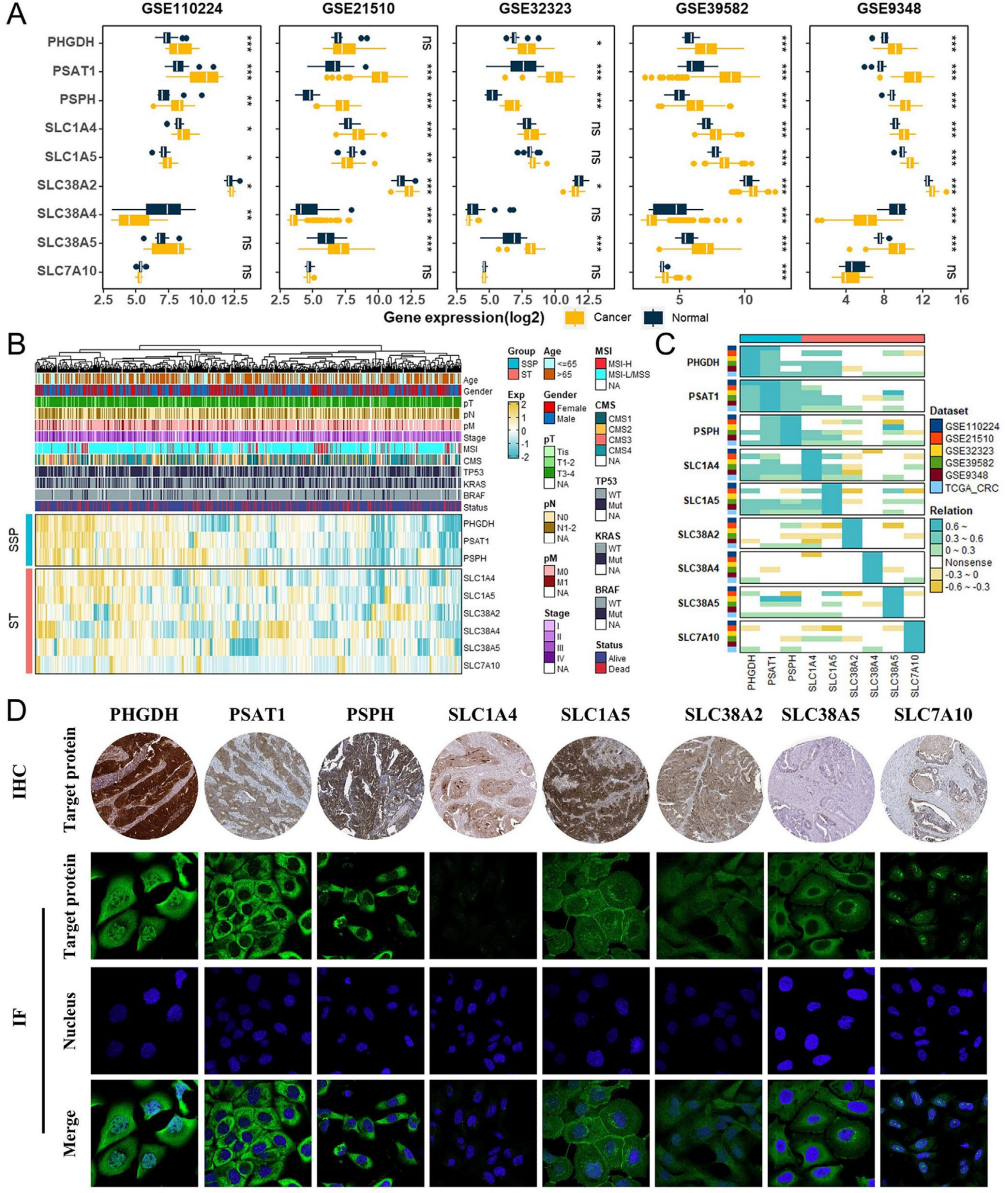
Expression patterns of SMGs in CRC. (A) Box plots depicting the expression levels of SMGs in tumour and normal tissues, based on the GEO datasets. (B) Heatmap integrating clinical annotations and mRNA expression of SMGs in CRC patients from the TCGA dataset. (C) Correlation analyses of SMGs in CRC. (D) Expression distribution of SMGs in CRC based on IHC (top) and IF (bottom) results from the HPA database. Note that SLC38A4 data is not available in the HPA database.

Given serine's established role as a critical exogenous nutrient for CRC proliferation [2], and our pan‐cancer findings confirming the dysregulation of SMGs in CRC (Figures 1 and S2A–C), we selected this malignancy for focused investigation. To validate the role of SMGs in CRC, we examined their expression across multiple GEO datasets. The results showed that PSAT1 and PSPH were consistently upregulated in tumour tissues in five GEO datasets, while PHGDH, SLC1A4 and SLC38A5 were upregulated in four datasets. In contrast, the expression patterns of SLC1A5, SLC7A10, SLC38A2 and SLC38A4 varied across datasets (Figure 2A).

We further analysed the distribution of SMGs in TCGA‐CRC based on various clinical features, including age, gender, TNM stage, microsatellite instability (MSI) status, molecular subtype and TP53/KRAS/BRAF mutation status (Figures 2B and S7A–K). Given that metastasis is a major factor contributing to poor prognosis in CRC patients, we specifically examined the association between these genes and N stage and M stage. Patients exhibiting high PSAT1 and SLC1A5 expression were more likely to develop lymph node metastasis, while high PHGDH, PSPH and SLC1A5 expression were associated with distant metastasis (Figures 2B and S7D–F). Additional analysis using GEO datasets revealed that PHGDH, PSAT1, PSPH, SLC1A4, SLC1A5 and SLC38A4 were highly expressed in both primary and metastatic CRC tissues compared to normal tissues (Figure S8A,B). However, no significant differences were observed between primary and metastatic tumour cell lines derived in CCLE (Figure S8C). Correlation analyses showed significant associations between the expression of PHGDH, PSAT1, PSPH, SLC1A4 and SLC1A5 in both CRC tissue samples and cell lines (Figures 2C and S8D). To explore the roles of SMGs in treatment response, we analysed six GEO datasets from patients treated with fluorouracil‐based ACT alone and three datasets with additional bevacizumab. The results showed that PHGDH, PSAT1, SLC38A4 and SLC38A2 exhibited differential expression in a few datasets (Figure S9A–I).

Finally, using the HPA database, we examined the expression and subcellular localization of SMGs in CRC (Figure 2D). IHC and immunofluorescence results indicated that PHGDH was localised in the cytoplasm and nucleus, while PSAT1 and PSPH were primarily cytoplasmic. Interestingly, as serine transmembrane transporters, only SLC1A5 was localised on the cell membrane and cytoplasm, while other genes were primarily expressed in the cytoplasm (Figure 2D). These findings suggest that the aberrant expression of SMGs, particularly key enzymes of the SSP, is closely associated with tumour progression in CRC.

### 3.3 Single‐Cell Profiling Unveils the Cellular Landscape of SMGs in CRC

**FIGURE 3:**
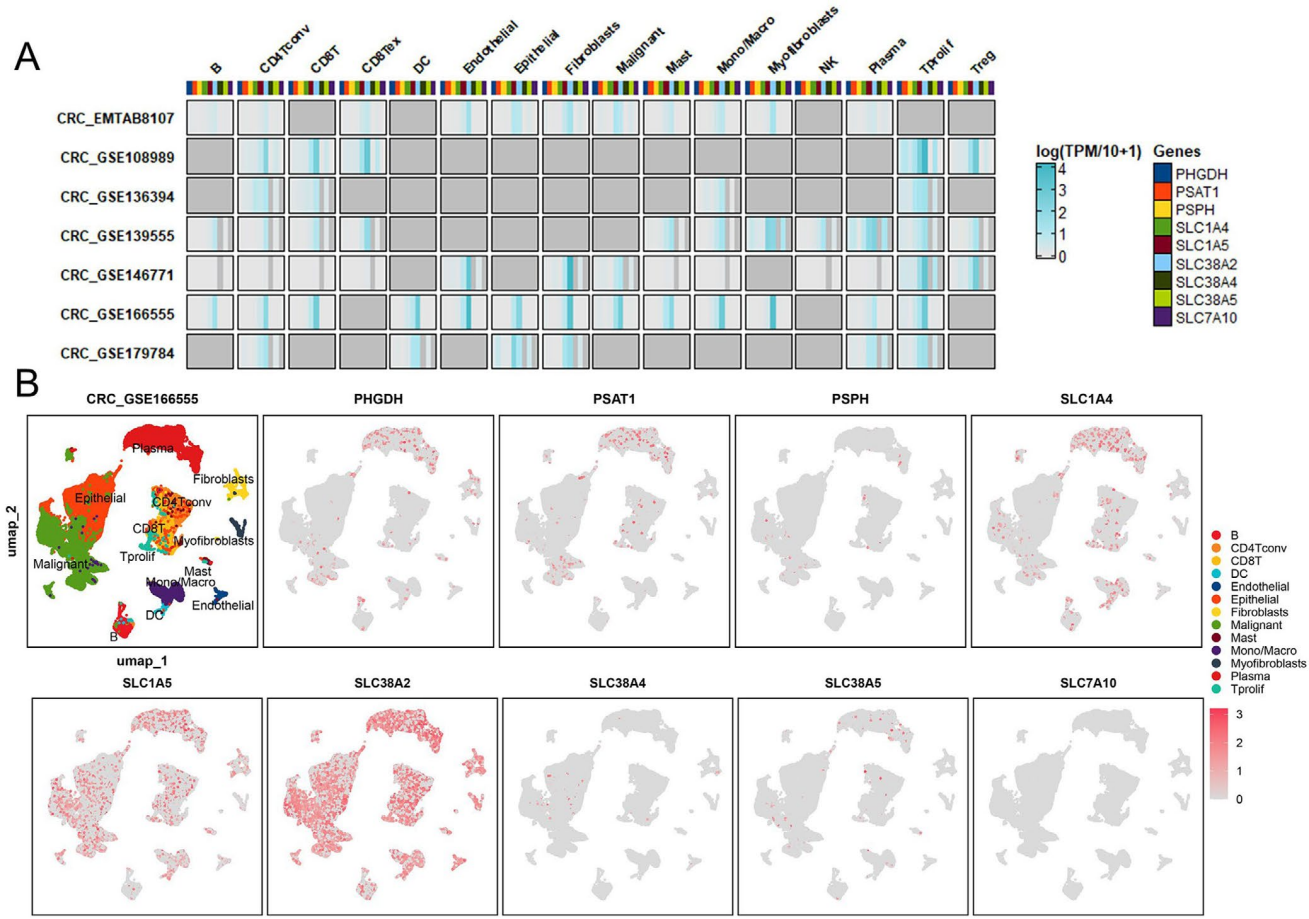
Single‐cell analyses of SMGs in different cell types in CRC. (A) Heatmap displaying the average expression levels of SMGs across various cell types in multiple single‐cell CRC datasets. (B) Scatter plots showing the average expression levels of SMGs in different cell types within the GSE166555 dataset.

To gain deeper insights into the cellular expression patterns of SMGs within tumour tissues, we conducted single‐cell level investigations using the TISCH2 database. The heatmap results showed that SMGs were broadly expressed across various tumour‐infiltrating cell types, including immune, stromal and malignant compartments, with particularly high expression in proliferating T cells (Tprolif), fibroblasts and regulatory myeloid cells (Treg/mono/macro) (Figure 3A). UMAP plots further demonstrated that in the microenvironment of CRC_GSE166555, PHGDH, PSAT1 and SLC1A4 were mainly expressed in plasma cells and malignant cells, SLC1A5 and SLC38A2 were widely expressed across nearly all cell types, while PSPH, SLC38A4 and SLC38A5 were restricted to a limited number of cells, and SLC7A10 was almost undetectable (Figure 3B). This provides a foundation for a more comprehensive understanding of the potential roles of SMGs in tumour development and cellular heterogeneity.

### 3.4 Prognostic Significance of SMGs in CRC

**FIGURE 4:**
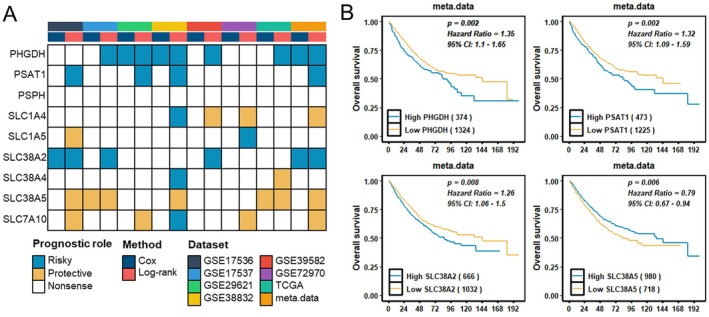
Prognostic value of SMGs in different CRC datasets. (A) Heatmap illustrating the associations between SMGs expression levels and overall survival, as determined by univariate Cox regression and Kaplan–Meier models. Blue represents risky factors, while yellow indicates protective factors. (B) Kaplan–Meier survival analyses of PHGDH, PSAT1, SLC38A2 and SLC38A5 in CRC meta.data.

Next, we explored the associations between the expression of SMGs and CRC prognosis using Cox regression analysis and Log‐rank tests across multiple CRC datasets. Elevated expression of PHGDH, PSAT1 and SLC38A2 was associated with poor prognosis, whereas low SLC38A5 expression predicted worse outcomes (Figure 4A). We further stratified CRC patients to evaluate the prognostic value of PHGDH expression across various clinical subgroups. Notably, PHGDH expression was significantly correlated with prognosis across multiple clinical subgroups, including female patients, T1‐2 and T3‐4 stages, N1‐2, M0, stage I/II and III/IV, MSI‐low (MSI‐L)/microsatellite stable (MSS), MSI‐high (MSI‐H), as well as tumours harbouring TP53 or KRAS mutations (Figure S10A–J). Interestingly, high PHGDH expression predicted better prognosis in the MSI‐H subgroup (Figure S10G), while it was linked to worse outcomes in other groups, including the MSI‐L/MSS group (Figure S10A–J).

### 3.5 Functional Enrichment Analysis Elucidates Molecular Mechanisms of SMGs

**FIGURE 5:**
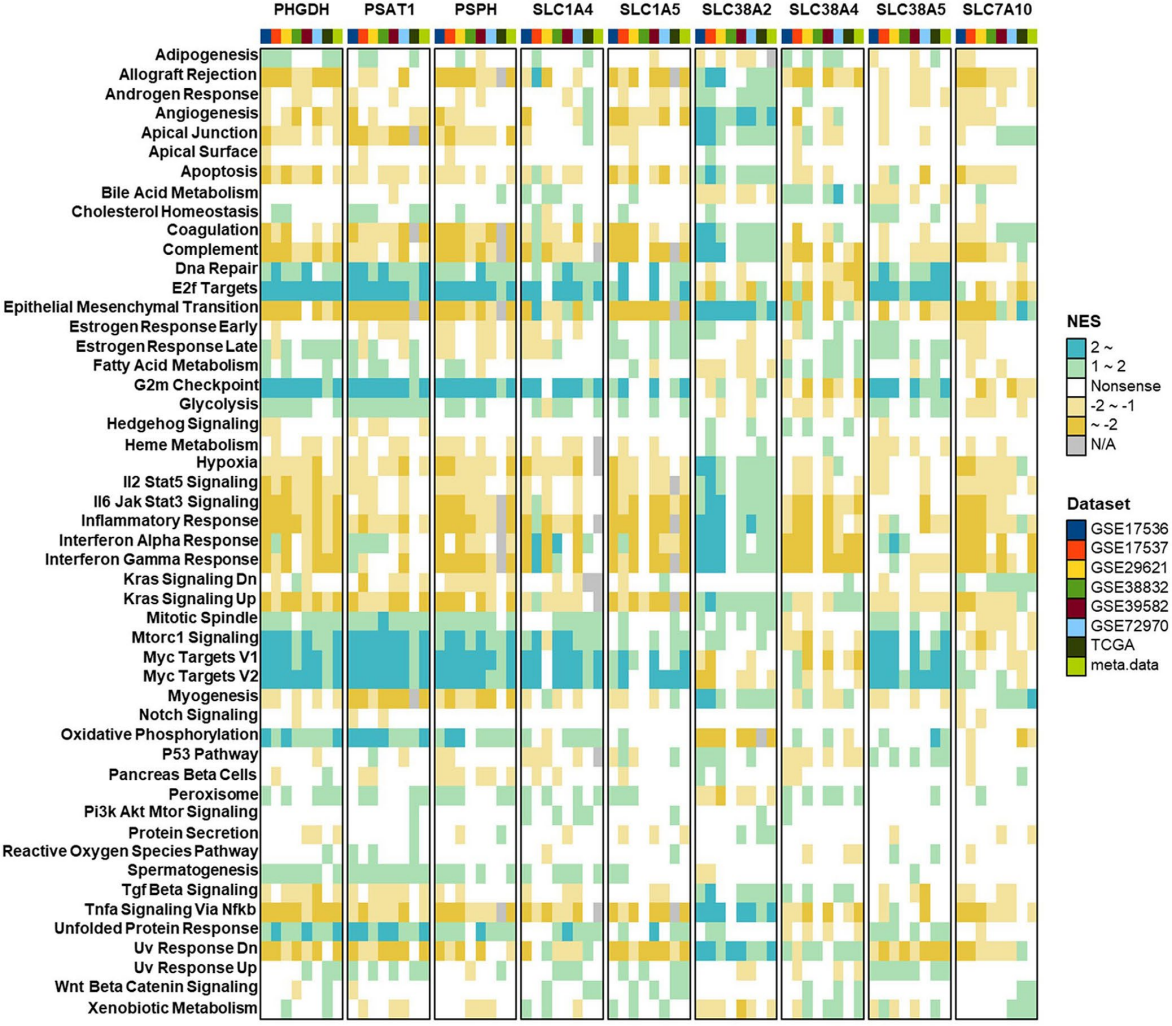
GSEA Analyses of SMGs Reveal Potential Biological Functions in CRC. Enrichment analyses of hallmark gene signature pathways between high and low SMGs expression in various CRC datasets. The normalised enrichment score (NES) represents the enrichment score used in the GSEA algorithm.

Given the aberrant expression of SMGs in CRC and its impact on cancer prognosis, we subsequently conducted GSEA to further investigate the potential molecular mechanisms underlying their functions (Figure 5). The results showed that PHGDH, PSAT1, PSPH, SLC1A5 and SLC38A5 were positively associated with cell proliferation‐related pathways, including DNA repair, E2F targets, G2M checkpoint, mTORC1 signalling and MYC targets V1/V2. In contrast, SLC38A2 was mainly associated with epithelial‐mesenchymal transition (EMT), angiogenesis and inflammation‐related pathways (e.g., allograft rejection, IL2‐STAT5 signalling, IL6‐JAK‐STAT3 signalling, inflammatory response and interferon alpha/gamma response), implying the potential involvement of SLC38A2 in regulating the tumour immune microenvironment. Given the subgroup‐specific prognostic effects of PHGDH across MSI and KRAS mutation subgroups, we further examined its pathway associations within these contexts. Regardless of MSI status, PHGDH was positively correlated with cell proliferation pathway genes. However, in the MSI‐H subgroup, PHGDH was mainly negatively correlated with inflammation‐related pathways (e.g., allograft rejection, interferon alpha/gamma response, complement and IL6‐JAK‐STAT3 signalling) (Figure S11A,B). A similar pattern was observed in KRAS‐stratified analysis: PHGDH was positively correlated with proliferative pathways in both KRAS‐mutant and wild‐type groups, while it showed a negative correlation with inflammation‐related pathways specifically in KRAS wild‐type tumours (Figure S11C,D). To complement these findings, we utilised the DepMap dataset, a resource offering genome‐wide CRISPR‐Cas9 screens to uncover gene dependencies across human cancer cell lines [47, 48]. Functional screening revealed that knockout of PHGDH, PSAT1, SLC1A5 and SLC38A2 significantly impaired the survival of CRC cell lines, including those harbouring TP53, KRAS or BRAF mutations (Figure S12A–D).

### 3.6 Association Between SMGs and the Tumour Immune Microenvironment

**FIGURE 6:**
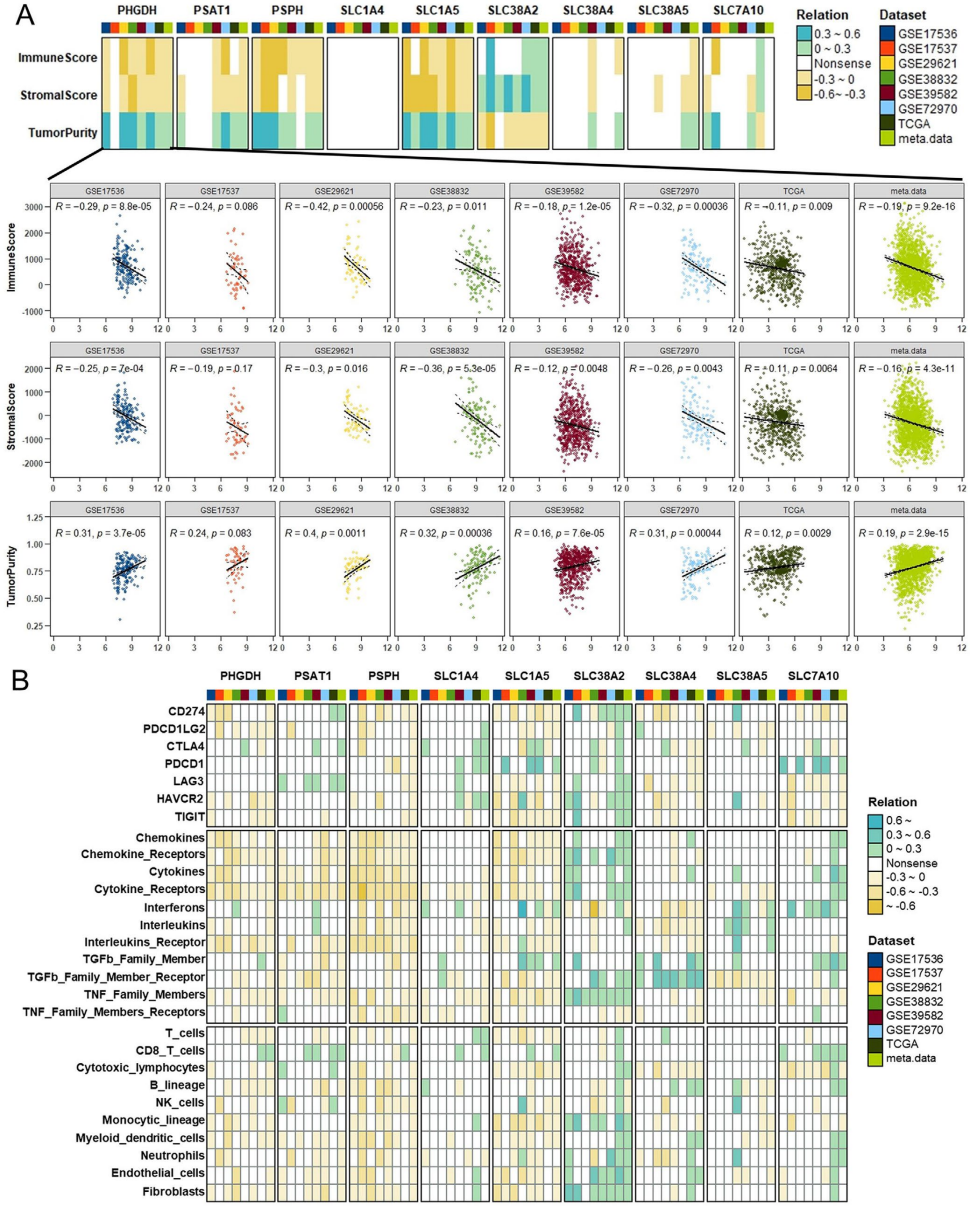
Correlation between SMGs and TME in CRC. (A) The heatmap (top) showing the correlation between SMGs and ImmuneScore, StromalScore and tumour purity across various CRC datasets. Scatter plots (bottom) illustrating the correlation of PHGDH expression with ImmuneScore, StromalScore and tumour purity. (B) Correlation analyses of SMGs with immune checkpoints (top), immune cytokines score (middle) and immune cells (bottom) across different CRC datasets.

To delve deeper into the relationship between SMGs and the tumour immune microenvironment, we initially calculated ImmuneScore, StromalScore and tumour purity across various CRC datasets and assessed their associations with SMG expression levels (Figure 6A). Correlation analyses showed that PHGDH, PSAT1, PSPH and SLC1A5 were negatively correlated with both ImmuneScore and StromalScore, while SLC38A2 exhibited a positive correlation with these scores (Figure 6A). Given that immune checkpoint inhibitor therapy has been shown to improve overall survival in multiple cancer types [49], including CRC, we next explored the associations between SMG expressions and immune checkpoint genes (Figure 6B). PHGDH and PSPH were negatively correlated with multiple immune checkpoints, while PSAT1, SLC1A4 and SLC38A2 displayed positive correlations with various checkpoint molecules (Figure 6B). We also explored the relationship between SMG expressions and immune cytokine signatures. PHGDH, PSAT1, PSPH and SLC1A5 were negatively correlated with scores for chemokines, chemokine receptors, cytokines, cytokine receptors and interleukin receptors, while SLC38A2 was positively correlated with TNF family members (Figure 6B). Furthermore, using the MCP‐counter algorithm, we found that PHGDH, PSAT1, PSPH and SLC1A5 correlated weakly with immune cells, while SLC38A2 was positively correlated with monocytic lineage, neutrophils and fibroblasts (Figure 6B). Finally, we analysed the correlation between PHGDH expression and immune‐related features—including ImmuneScore, StromalScore, immune checkpoints, cytokine signatures and immune cell infiltration—in CRC patients stratified by MSI status and KRAS mutation. The results demonstrated that these correlations were stronger in MSI‐H and KRAS‐mutant subgroups (Figure S13A–D).

### 3.7 Expression Patterns of PHGDH, SLC1A5 and SLC38A2 in Real‐World Cohorts

**FIGURE 7:**
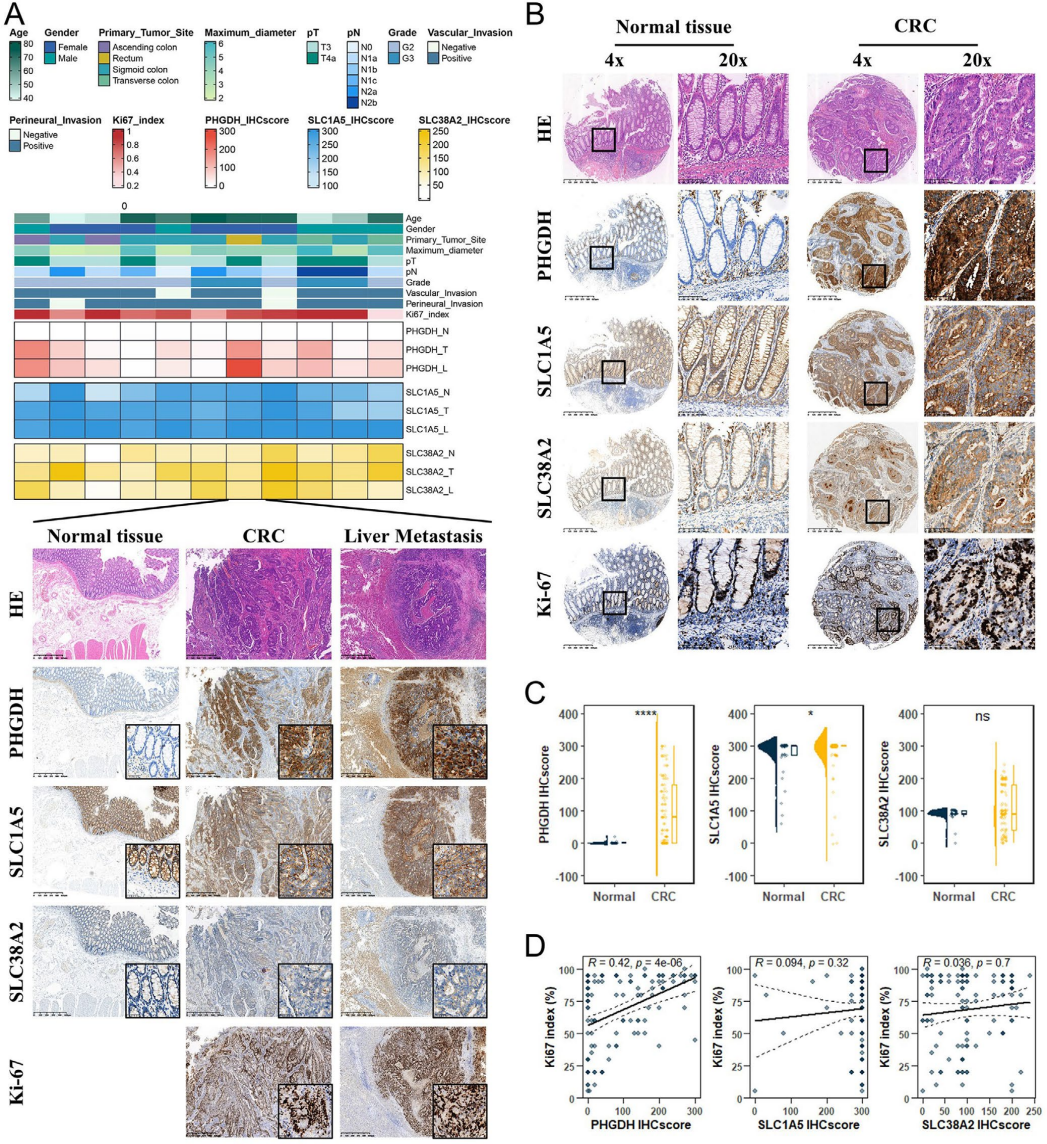
Differential expression of PHGDH, SLC1A5 and SLC38A2 in CRC and metastasis across real‐world Cohorts. Heatmap (top) displaying clinical information and IHCscore for cohort 1; representative immunohistochemistry images (bottom) showing the expression distribution of PHGDH, SLC1A5 and SLC38A2 in normal tissues, primary CRC tumours and matched liver metastases. Expression distribution of PHGDH, SLC1A5 and SLC38A2 in CRC tissue microarrays from cohort 2. Statistical analyses of PHGDH, SLC1A5 and SLC38A2 expression in tissue microarrays from cohort 2. (D) Scatter plots illustrating the correlations between the expression of PHGDH, SLC1A5 and SLC38A2 and Ki‐67 proliferation index in CRC samples from in cohort 2.

Considering the elevated expression of PHGDH, SLC1A5 and SLC38A2 in CRC (Figures 1, 2A, S2A–D, S3 and S8A) and their association with tumour proliferation (Figures 5, S12A–D), metastasis (Figures 2B, S8A,B) and patient prognosis (Figure 4A), we selected these genes for validation in real‐world patient cohorts. We first performed staining for PHGDH, SLC1A5 and SLC38A2 in 11 matched samples from cohort 1, including normal colorectal tissues, primary CRC tissues and corresponding liver metastases (Figure 7A). At low magnification, PHGDH expression in normal colorectal tissue was primarily localised to the lamina propria and germinal centres (GCs) of lymphoid nodules, with minimal expression in the intestinal epithelium (Figures 7A and S14A). In contrast, SLC1A5 and SLC38A2 were predominantly expressed in the intestinal epithelium, lamina propria and GCs of lymphoid nodules, with SLC1A5 showing relatively higher expression and SLC38A2 lower expression (Figures 7A and S14A). In CRC tissues, PHGDH, SLC1A5 and SLC38A2 were mainly expressed in tumour cells and the GCs of mature TLSs (Figures 7A and S14A). In liver metastases, PHGDH and SLC38A2 were expressed in tumour cells, surrounding hepatocytes and bile duct cells. Notably, SLC1A5 was restricted to tumour cells, with no expression in hepatocytes or bile duct epithelium (Figures 7A and S14A). Higher magnification analysis revealed consistent subcellular localisation across tissue types. PHGDH and SLC38A2 expression was cytoplasmic, while SLC1A5 was present on both the cell membrane and in the cytoplasm (Figures 7A and S14A). Consistent with cohort 1, the expression patterns of PHGDH, SLC1A5 and SLC38A2 in normal and tumour tissues from cohort 2 were identical for all three markers (Figure 7B).

### 3.8 Upregulation of PHGDH, SLC1A5 and SLC38A2 in Colorectal Cancer and Metastatic Tissues

To investigate the expression patterns of PHGDH, SLC1A5 and SLC38A2 in tumour cells, we first compared their levels among normal epithelium, primary CRC and metastatic tumours in cohort 1. Compared to normal controls, PHGDH was highly expressed in both primary and metastatic CRC, with no significant difference observed between the two tumour types (Figures 7A and S14B). SLC1A5 was highly expressed in liver metastases, and SLC38A2 was mainly highly expressed in primary CRC (Figures 7A and S14B). In the tissue microarray from cohort 2, PHGDH and SLC1A5 were highly expressed in CRC (Figure 7B,C), while SLC38A2 showed no significant difference in expression between normal tissues and CRC (Figure 7B,C). Correlation analyses in cohort 2 further revealed that PHGDH expression was positively correlated with the Ki67 index, and SLC38A2 showed a weak correlation with both PHGDH and SLC1A5 (Figures 7D and S14C). We also assessed the associations between gene expression and clinicopathological features, including age, gender, tumour location, tumour size, histological differentiation and TNM stage. PHGDH expression was significantly associated with tumour size and N stage, while SLC1A5 expression was correlated with T stage (S15A–H). In contrast, SLC38A2 expression showed no significant association with these clinical parameters (S15A–H). These findings confirm that these genes display varying degrees of overexpression in CRC, consistent with the earlier bioinformatics results. Moreover, real‐world cohort analysis revealed that PHGDH was significantly overexpressed in both primary CRC and liver metastases, and its expression positively correlated with the Ki67 index, tumour size and N stage, suggesting its potential role as a key metabolic driver in CRC progression.

## 4 Discussion

Cancer cells maintain intracellular serine levels through endogenous synthesis and/or exogenous uptake, which in turn fuels key metabolic pathways such as the serine‐glycine one‐carbon pathway, tricarboxylic acid cycle and lactic acid and lipid synthesis, thereby promoting robust tumour growth and proliferation [5, 6, 7, 8, 50]. The SSP originates from the glycolytic or gluconeogenic intermediate 3‐PG, which is converted to serine through a three‐step enzymatic cascade involving PHGDH, PSAT1 and PSPH. Inhibition of these key enzymes has been shown to suppress the growth of tumours such as lymphoma [51], CRC [52, 53] and breast cancer [54]. Beyond de novo synthesis, tumour cells acquire extracellular serine via specific transporters, whose silencing impairs cancer cell growth [15]. Serine deprivation also inhibits CRC cell proliferation in vitro and reduces xenograft tumour growth in vivo [2, 5, 55]. Recent preclinical studies further demonstrate that targeting serine metabolism—through blockade of endogenous synthesis, restriction of dietary serine and glycine or a combination of both—elicits potent antitumor effects [3, 56, 57]. However, it remains largely unclear which subsets of tumour patients would benefit most from targeting serine metabolism, and whether interventions should focus on inhibiting synthesis, uptake or both. Addressing this knowledge gap forms the central rationale for our investigation.

Across diverse cancer types, SMGs exhibit distinct expression profiles, reflecting their molecular heterogeneity and underscoring their roles as critical regulators of tumour progression. These patterns lay the groundwork for a deeper understanding of the multifaceted contributions of SMGs to cancer development. Focusing on CRC, our analyses—integrating TCGA and GTEx data, as well as paired and unpaired samples from TCGA—consistently reveal upregulation of PHGDH, PSAT1, PSPH, SLC1A4, SLC1A5 and SLC38A5. These findings suggest a potential oncogenic role for these genes, aligning with prior studies showing that inhibition of serine biosynthesis or limitation of exogenous serine uptake can suppress CRC growth [2, 5, 55, 58, 59]. Beyond expression profiling, our pan‐cancer analysis uncovers a spectrum of genetic and epigenetic alterations in SMGs, including mutations, CNVs, DNA methylation changes and RNA modifications. These alterations highlight the intricate regulatory landscape of SMGs and offer critical insights into the mechanisms driving their dysregulation during tumourigenesis.

The abnormal expression of SMGs across various cancers prompted us to select a specific type of cancer for in‐depth analysis. Given the significant heterogeneity in molecular subtypes, clinical outcomes and therapeutic responses observed in CRC, we chose it as the model for our experimental validation [60, 61, 62, 63]. Using CRC as a model, Maddocks et al. demonstrated that cancer cells rapidly consume exogenous serine, and its deprivation activates the SSP while suppressing aerobic glycolysis, resulting in increased TCA cycle flux [2]. Consistent with these findings, SMGs were aberrantly expressed in CRC across the TCGA pan‐cancer dataset and validated in multiple independent CRC cohorts. Notably, high PHGDH, PSAT1, PSPH and SLC1A5 expression were linked to metastasis, and several SMGs were significantly associated with CRC prognosis. For instance, elevated PHGDH expression consistently predicted poorer outcomes across both early (stage I/II) and advanced (stage III/IV) disease, highlighting its potential as a marker of disease progression.

The identification of SMGs as potential prognostic indicators motivated us to explore their mechanistic roles in cancer prognosis. Functional enrichment analysis in our study revealed that dysregulation of these genes is intricately linked to key biological processes and oncogenic pathways. Serine, as a major contributor of one‐carbon units, supports de novo synthesis of purines and deoxythymidine—crucial for DNA replication during cell proliferation [64]. Notably, genes such as PHGDH, PSAT1, PSPH, SLC1A4, SLC1A5 and SLC38A5 were found to be associated with cell proliferation‐related pathways. Moreover, CRISPR knockout data from the DepMap database in CRC cell lines revealed that knocking out genes such as SLC1A5, PSAT1 and PHGDH profoundly inhibits cell growth, further underscoring their essential roles in CRC. Equally compelling is the association between SMGs and immune‐related pathways. Further evaluation of the correlation between these genes and immune checkpoints, cytokine scores and immune cell populations provides valuable insights into the complex interplay between serine metabolism and both antitumor and protumour immune activities. The TNF superfamily plays critical roles in immune responses, cell death and proliferation by activating diverse intracellular signalling cascades [65], and SLC38A2 expression showed a positive correlation with several TNF family members. According to gene expression profiles, the consensus molecular subtypes (CMS) classification divides CRC into four distinct subtypes [66]. Interestingly, SLC38A2 which is significantly positively correlated with EMT, is predominantly expressed in the CMS4 subtype, which is characterised by TGF‐β signalling, EMT activation and resistance to chemotherapy [66].

The TME is composed of various cellular components, including innate immune cells, adaptive immune cells, mast cells, myeloid‐derived suppressor cells, cancer cells and surrounding stromal cells [6, 67]. Given that transcriptomic data are derived from whole tumour samples, and considering the high heterogeneity of CRC, we conducted a more in‐depth analysis of SMGs at the single‐cell level. SMGs are not only expressed in tumour cells but also in other cell types, including plasma cells, monocytes and macrophages. In plasma cells, serine plays a crucial role beyond serving as a substrate for antibody and nucleic acid synthesis; it also supports energy metabolism and antioxidant defence mechanisms [6, 68]. Additionally, M2‐type tumour‐associated macrophages exhibit a higher capacity for glucose uptake from the tumour milieu, indicating that they are significant contributors to serine production derived from tumour glycolysis [6, 69]. The balance between the oncogenic and antitumour immune functions of serine metabolism is influenced by the abundance and activation state of different cell types, as well as the expression profiles of various immune mediators and regulators within the TME [67]. This dynamic interplay may also explain the varying prognostic implications of PHGDH expression in MSI‐H and MSI‐L/MSS CRC patients. MSI‐H tumours are typically characterised by a higher mutation burden and increased neoantigen load, contributing to a more immunogenic tumour microenvironment [70, 71]. Single‐cell analysis revealed that PHGDH is predominantly expressed in plasma cells within the immune compartment. This observation was further validated by IHC staining, which demonstrated PHGDH expression in the GCs of mature TLSs—regions enriched with plasma cells. Within this context, PHGDH may support the activation and maintenance of anti‐tumour immune cell functions, especially those of plasma cells. In contrast, in the immunologically ‘cold’ MSI‐L/MSS tumours, high PHGDH expression appears to primarily promote tumour cell proliferation and may also enhance the activity of immunosuppressive cells, such as fibroblasts and M2 macrophages. This microenvironment‐dependent dual role suggests that the prognostic impact of PHGDH is shaped by a dynamic balance between its protumour metabolic functions and its support of immune‐mediated antitumour responses. This balance likely differs significantly between MSI statuses. Understanding this balance could offer new insights into how serine metabolism influences CRC progression and patient outcomes, potentially guiding more personalised therapeutic strategies.

By comparing the impact of SMGs on CRC metastasis, prognosis and cell viability, we selected PHGDH, SLC1A5 and SLC38A2 for further validation in two independent real‐world cohorts. Our results indicate that PHGDH is minimally expressed in normal tissues but highly expressed in tumour tissues. In contrast, although SLC1A5 and SLC38A2 showed statistically significant differences in certain datasets, their expression levels between normal intestinal epithelium and tumour cells were not markedly distinct. In terms of expression patterns, PHGDH exhibited heterogeneous expression, while SLC1A5 and SLC38A2 showed diffuse staining. Moreover, despite both functioning as serine transporters, SLC1A5 and SLC38A2 differ in their cellular localization: SLC1A5 is found in both the cell membrane and cytoplasm, whereas SLC38A2 is localised exclusively in the cytoplasm. This suggests that the uptake of extracellular serine is primarily mediated by SLC1A5. Further analysis of the expression differences of PHGDH, SLC1A5 and SLC38A2 between primary CRC and liver metastases revealed no significant differences in either expression intensity or pattern. In adjacent normal liver tissues, PHGDH and SLC38A2 were highly expressed in hepatocytes and bile duct cells, while SLC1A5 was barely detectable. Interestingly, in CRC liver metastases, SLC1A5 was diffusely overexpressed, suggesting its potential as a novel marker for CRC liver metastasis. Notably, the ‘high‐serine’ environment of the liver did not appear to influence the expression of PHGDH, SLC1A5 and SLC38A2 in CRC liver metastases. This finding underscores the complexity of serine metabolism in the context of cancer metastasis and suggests that SLC1A5 could be a critical player in the adaptation of CRC cells to metastatic sites.

The presence of intratumoral TLSs is associated with favourable clinical outcomes and enhanced response to immunotherapy in cancer [68, 72]. TLSs are characterised by an inner zone of CD20+ B cells surrounded by CD3+ T cells, resembling the lymphoid follicles of secondary lymphoid organs [72]. TLSs can be classified into immature TLS (iTLS) and mature TLS (mTLS). iTLS consists of aggregates of T and B cells with a few dendritic cells (DCs), while mTLS includes B‐cell zones that form primary follicles (PFL) or secondary follicles (SFL) with GCs [73, 74]. The formation of GCs in secondary lymphoid organs is crucial for generating high‐affinity, long‐lived plasma cells and memory B cells [75]. This process is regulated by antigen‐driven interactions between B cells and T follicular helper (TFH) cells. The presence of GCs in TLSs suggests that similar antigen recognition processes occur within these structures [29]. IHC analysis revealed that PHGDH, SLC1A5 and SLC38A2 are expressed in the GCs of TLSs in both normal and tumour colorectal tissues, suggesting that serine metabolism might play a critical role in GC formation. Notably, while PHGDH exhibits heterogeneous expression in tumour cells, its expression in the GCs of TLSs is more frequent and uniform. This difference hints at distinct regulatory mechanisms for PHGDH expression in tumour cells versus GCs. In summary, this detailed analysis not only underscores the significance of SMGs, particularly PHGDH and SLC1A5, in cancer biology but also lays the groundwork for unravelling the complex molecular mechanisms through which serine influences the tumour microenvironment and immune architecture.

While our study offers valuable insights into the roles of SMGs across cancers, particularly in CRC, several limitations warrant consideration. First, our findings are primarily based on bioinformatics analyses. Although we performed extensive validation in CRC samples, functional experiments are needed to establish causal relationships between SMGs and the observed phenotypes. Second, although we employed scRNA‐seq and IHC to examine the expression patterns of PHGDH, SLC1A5 and SLC38A2 within the TME, their precise roles in specific cell types remain unclear. Moreover, given the metabolic plasticity of cancer cells, PHGDH inhibition may trigger compensatory serine uptake via transporters. This highlights the potential need for dual‐targeting strategies that simultaneously block endogenous serine synthesis and exogenous uptake. Future work using preclinical CRC models is essential to validate these mechanisms and evaluate the therapeutic efficacy of such combination approaches. Finally, this study primarily focused on the prognostic relevance of individual SMGs. Integrating their expression profiles with clinical features—such as tumour stage, MSI status and common oncogenic mutations (e.g., TP53, KRAS, BRAF)—may help develop more robust and clinically applicable prognostic models.

In conclusion, our comprehensive study reveals the complex role of SMGs across multiple cancer types, highlighting their differential expression patterns, genetic variations and prognostic significance. These findings underscore the multifaceted involvement of serine metabolism in cancer biology. Specifically, we provided an in‐depth characterisation of the expression profiles of SMGs in CRC and examined their associations with clinical outcomes, functional pathways and the immune microenvironment. Together, these findings not only enhance our understanding of serine metabolism in CRC but also pave the way for developing targeted therapies and personalised treatment strategies aimed at disrupting serine metabolism in cancer cells.

## Author Contributions

**Anqi Li:** conceptualization (equal), visualization (equal), writing – review and editing (equal). **Qihui Wu:** supervision (equal), writing – original draft (lead), writing – review and editing (equal). **Yuanyuan Xu:** conceptualization (equal), validation (equal), writing – review and editing (equal). **Yijin Gu:** formal analysis (equal), investigation (equal), methodology (equal), writing – review and editing (equal). **Xuan Wang:** formal analysis (equal), writing – review and editing (equal). **Jiaxin Liu:** methodology (equal), supervision (equal), writing – review and editing (equal). **Yan Wang:** investigation (equal), methodology (equal). **Jialing Xie:** software (equal), writing – review and editing (equal). **Xiaodan Fu:** conceptualization (equal), project administration (equal), supervision (equal), writing – review and editing (equal). **Yimin Li:** conceptualization (lead), data curation (equal), formal analysis (lead), funding acquisition (lead), investigation (lead), methodology (equal), project administration (equal), supervision (equal), validation (equal), writing – review and editing (equal).

## Ethics Statement

The present study underwent thorough scrutiny and received approval from the Ethics Committee of Xiangya Hospital, Central South University and Ruijin Hospital, Shanghai Jiaotong University School of Medicine, ensuring adherence to the principles outlined in the Declaration of Helsinki.

## Conflicts of Interest

The authors declare no conflicts of interest.

## Supporting information


**Figures S1–S15.** Supporting Figures.

## Data Availability

No data is available.
