# Supplementary material for: Multi‐Omics Analysis and Real‐World Data Validation of Serine Metabolism‐Related Genes in Colorectal Cancer
Source: J Cell Mol Med. 2025 Jul 14;29(13):e70721. doi: 10.1111/jcmm.70721 (PMC12259393; doi:10.1111/jcmm.70721)
Supplement: Supplementary file 1 — Figures S1–S15. Supporting Figures. [file JCMM-29-e70721-s001.docx]

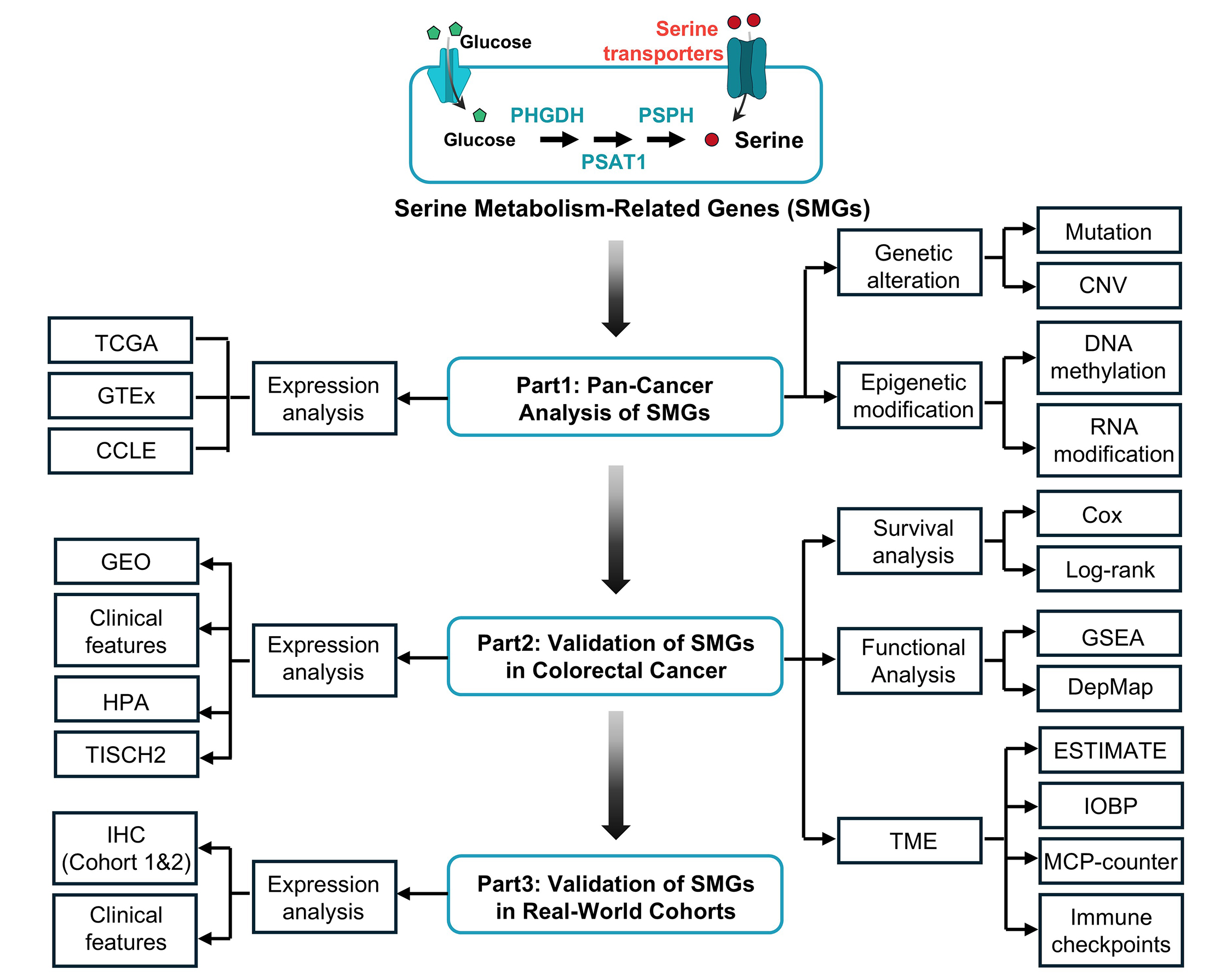


**Figure S1: A Visual Schematic Summarizing the Overall Workflow of the Study.**

**
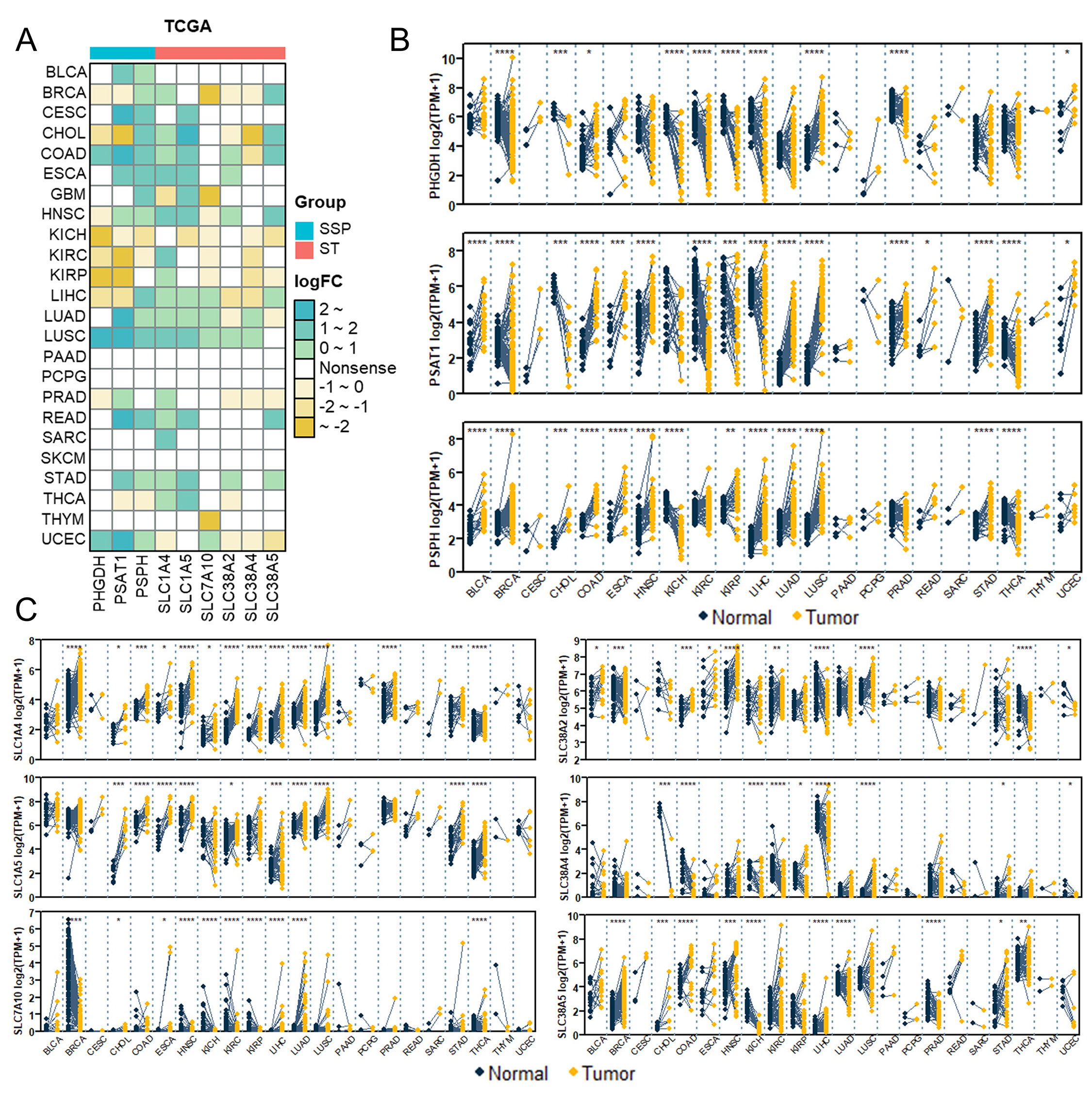
**

**Figure S2: Pan-Cancer Expression Analysis of SMGs.**

(A) Heatmap illustrating the distribution of SMGs across different cancers in TCGA.

(B, C) Box plots showing the expression distribution of key enzymes in the SSP (B) and ST (C) in paired tumor and normal tissues based on TCGA.

**
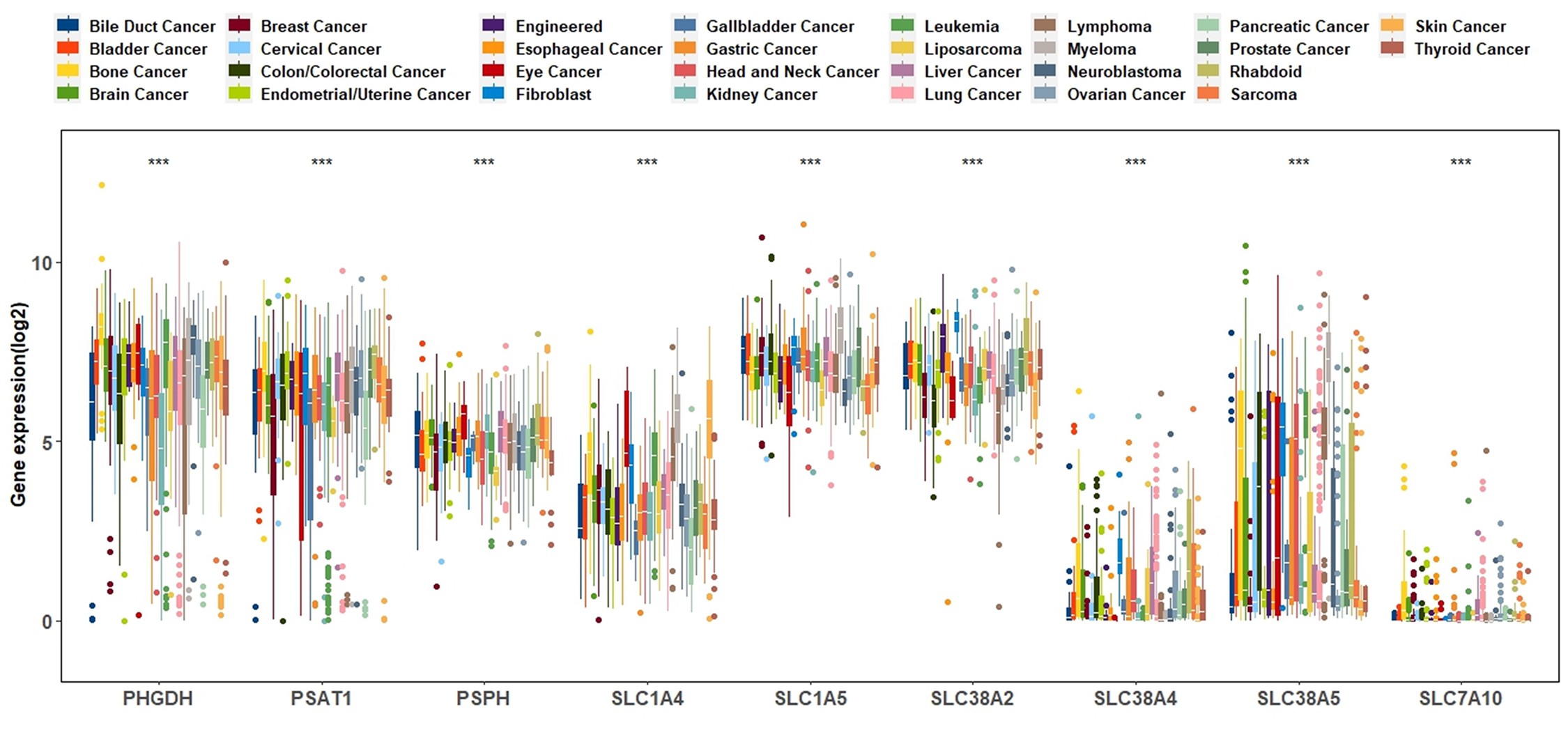
**

**Figure S3: Expression Distribution of SMGs in Various Cancer Cell Lines from the CCLE Database.**

**
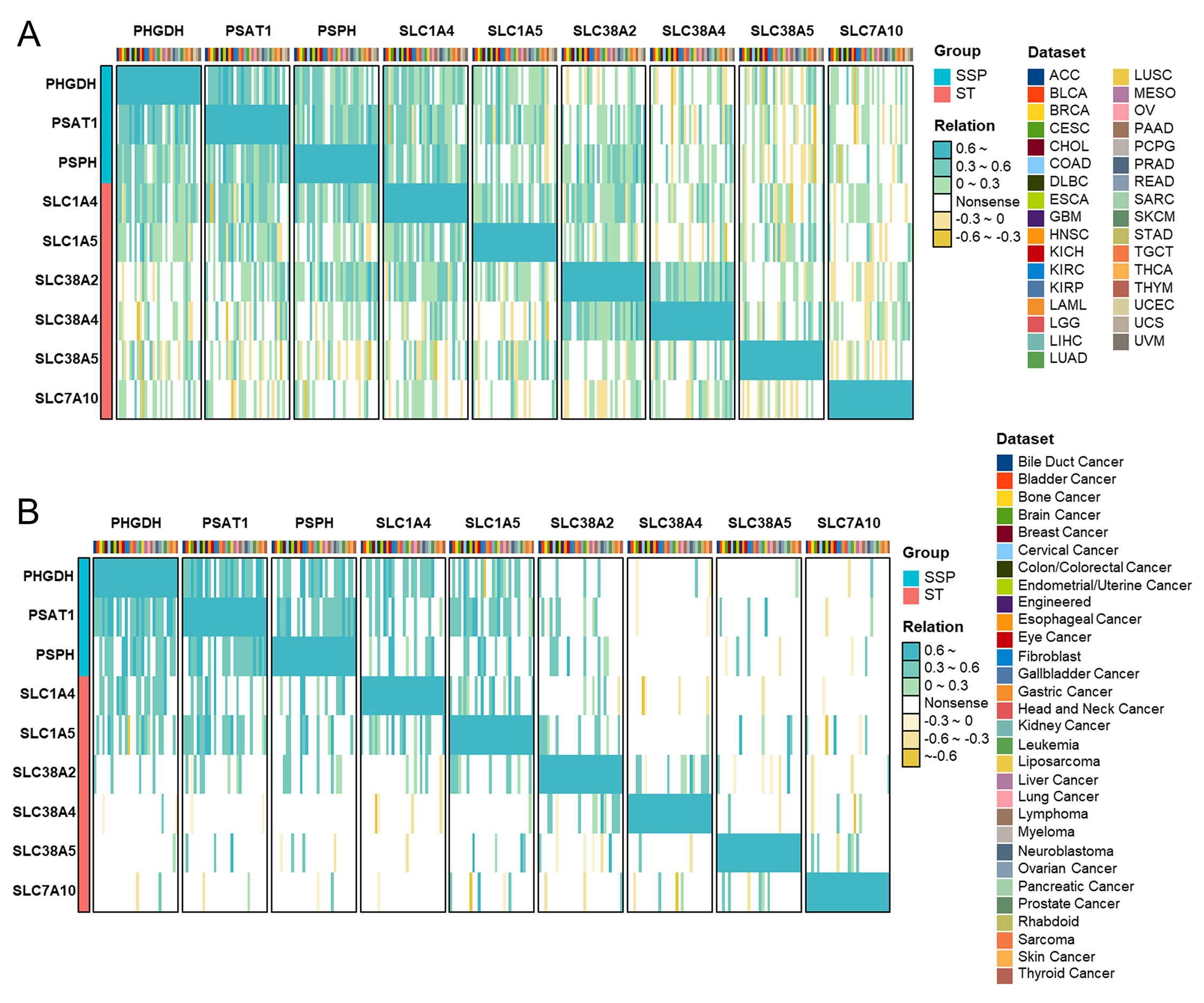
**

**Figure S4: Correlation Analysis of SMGs in Pan-Cancer and Cancer Cell Lines.**

(A, B) Heatmaps illustrating inter-gene correlations of SMGs in the TCGA (A) and CCLE (B) datasets.

**
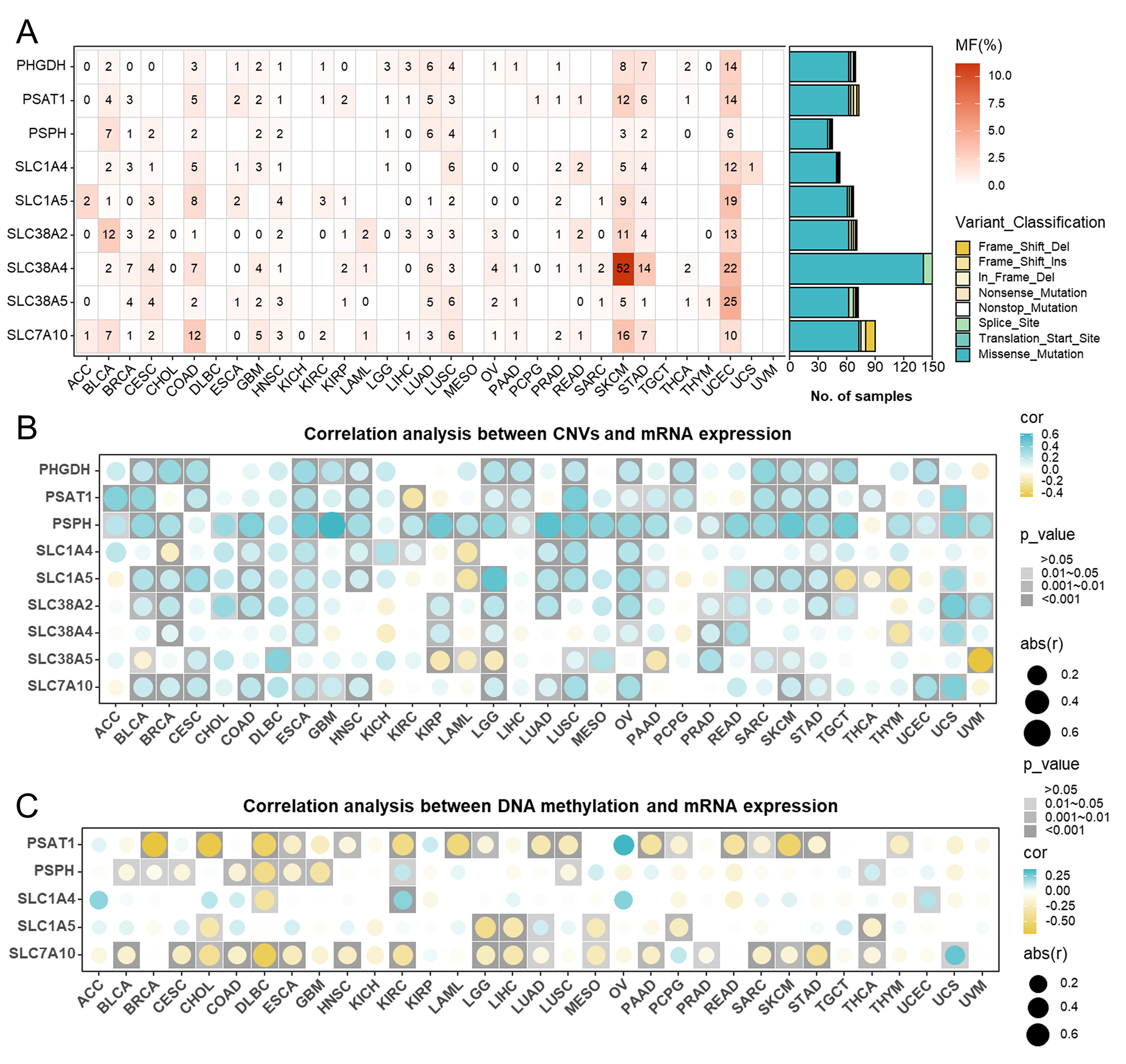
**

**Figure S5: Genetic Alterations and DNA Methylation of SMGs in Different Cancers.**

(A) Mutation frequencies of SMGs across various cancers. The heatmap on the left indicating the number of samples with mutated genes in specific cancer types. The bar graph on the right summarizes the types of single nucleotide variant observed in SMGs.

(B, C) Heatmaps showing the CNVs (B), DNA methylation levels (C), and their correlations with mRNA expression of SMGs in various tumors.

**
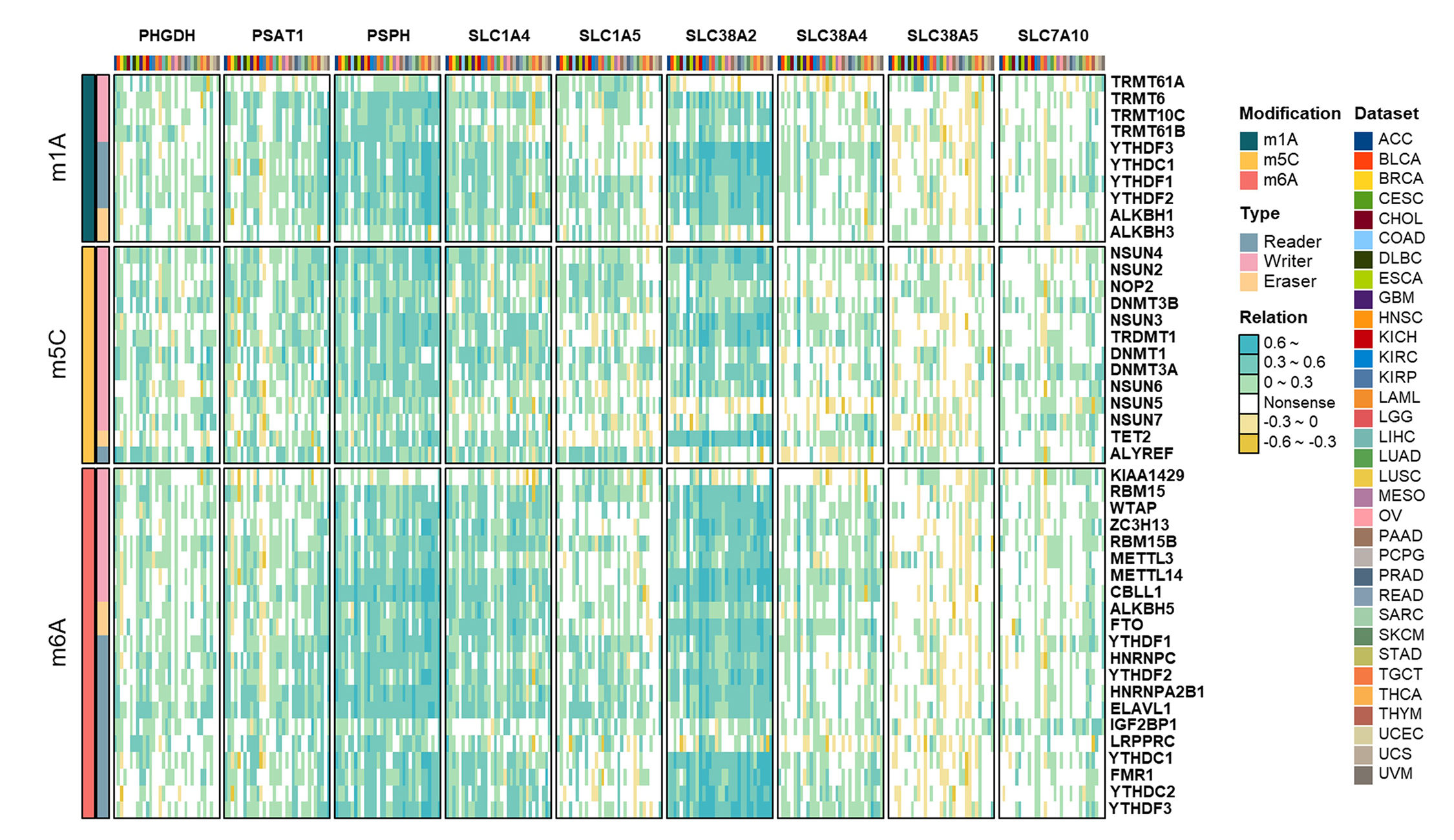
**

**Figure S6: Heatmap Illustrating the Relationship Between SMG Expression and RNA-modifying enzymes (m1A, m5C, m6A).**

**
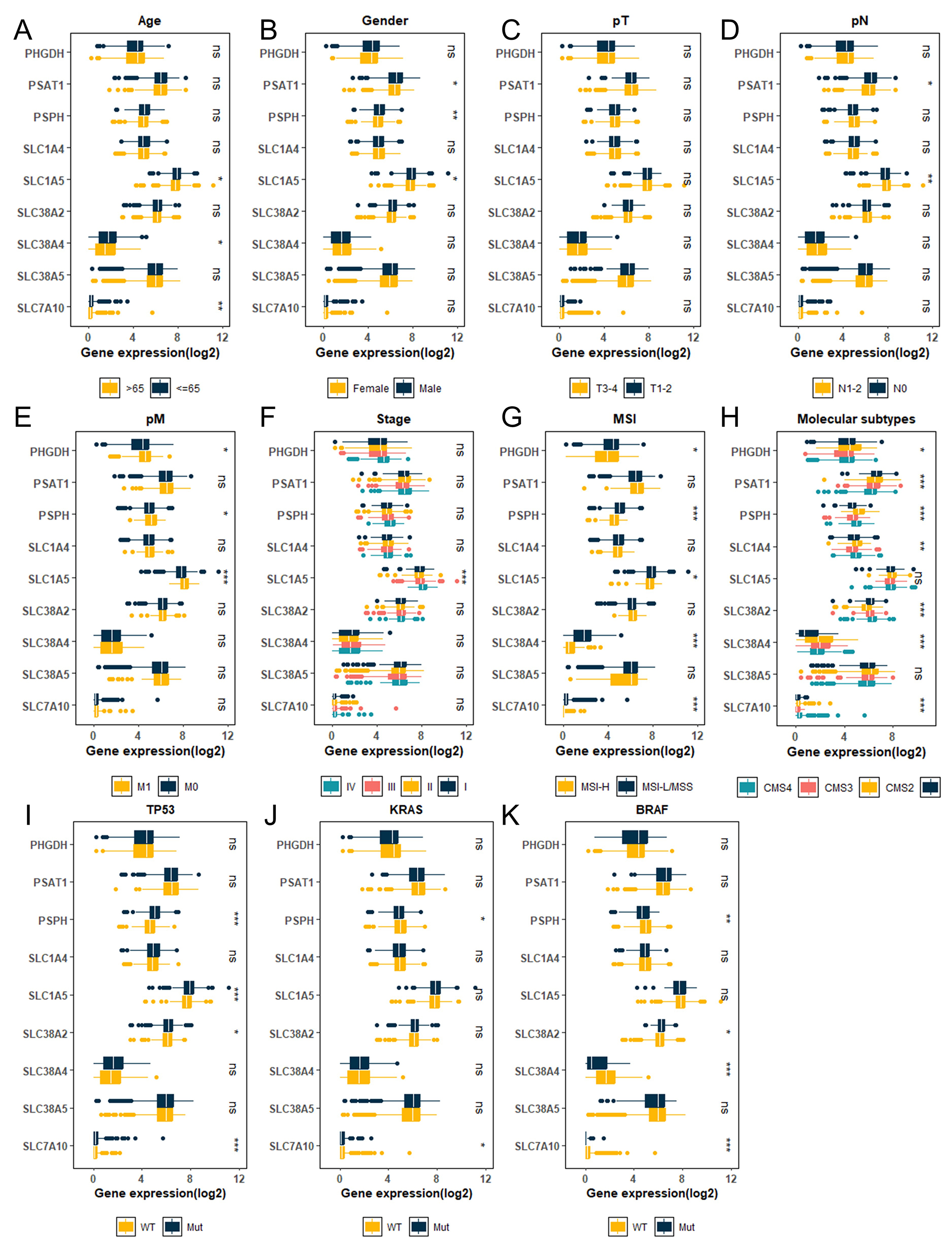
**

**Figure S7: Correlation Between SMG Expression and Clinical Features in CRC.**

(A-H) SMG expression levels stratified by various clinical features of CRC patients, including age (A), gender (B), T stage (C), N stage (D), M stage (E), TNM stage (F), MSI status (G), and CMS molecular subtype (H).

(I-K) SMG expression levels stratified by the mutational status of TP53 (I), KRAS (J), and BRAF (K) in CRC patients.

**
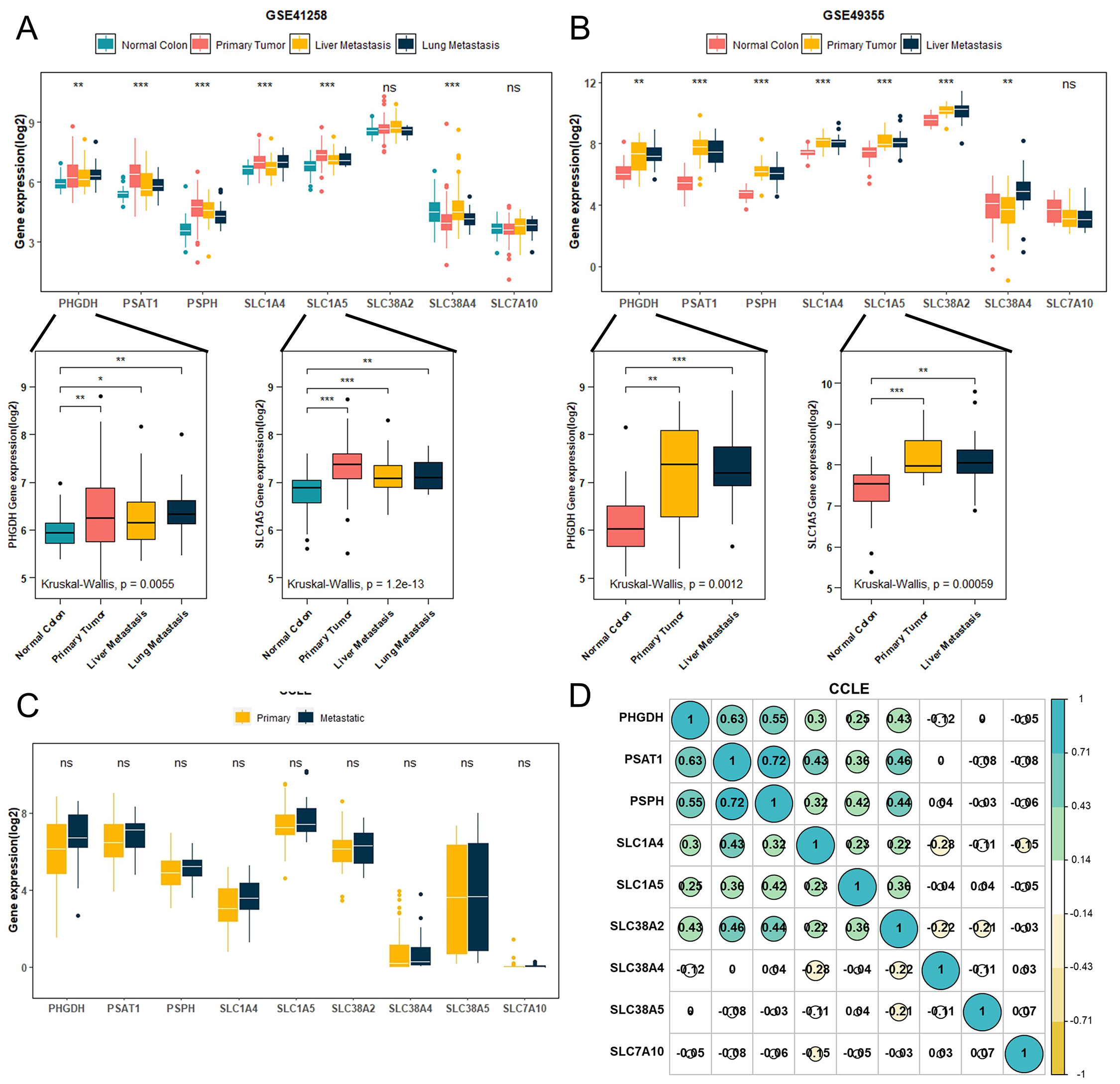
**

**Figure S8:** **Expression of SMGs in Primary and Metastatic CRC Tissues.**

(A, B) Box plots illustrating the distribution of SMG expression in normal tissues, primary CRC, and metastatic liver/lung tissues based on the GSE41258 (A) and GSE49355 (B) datasets.

(C) Expression distribution of SMGs in primary and metastatic CRC cell lines from the CCLE database.

(D) Correlation analyses of SMGs in CRC cell lines from the CCLE database.

**
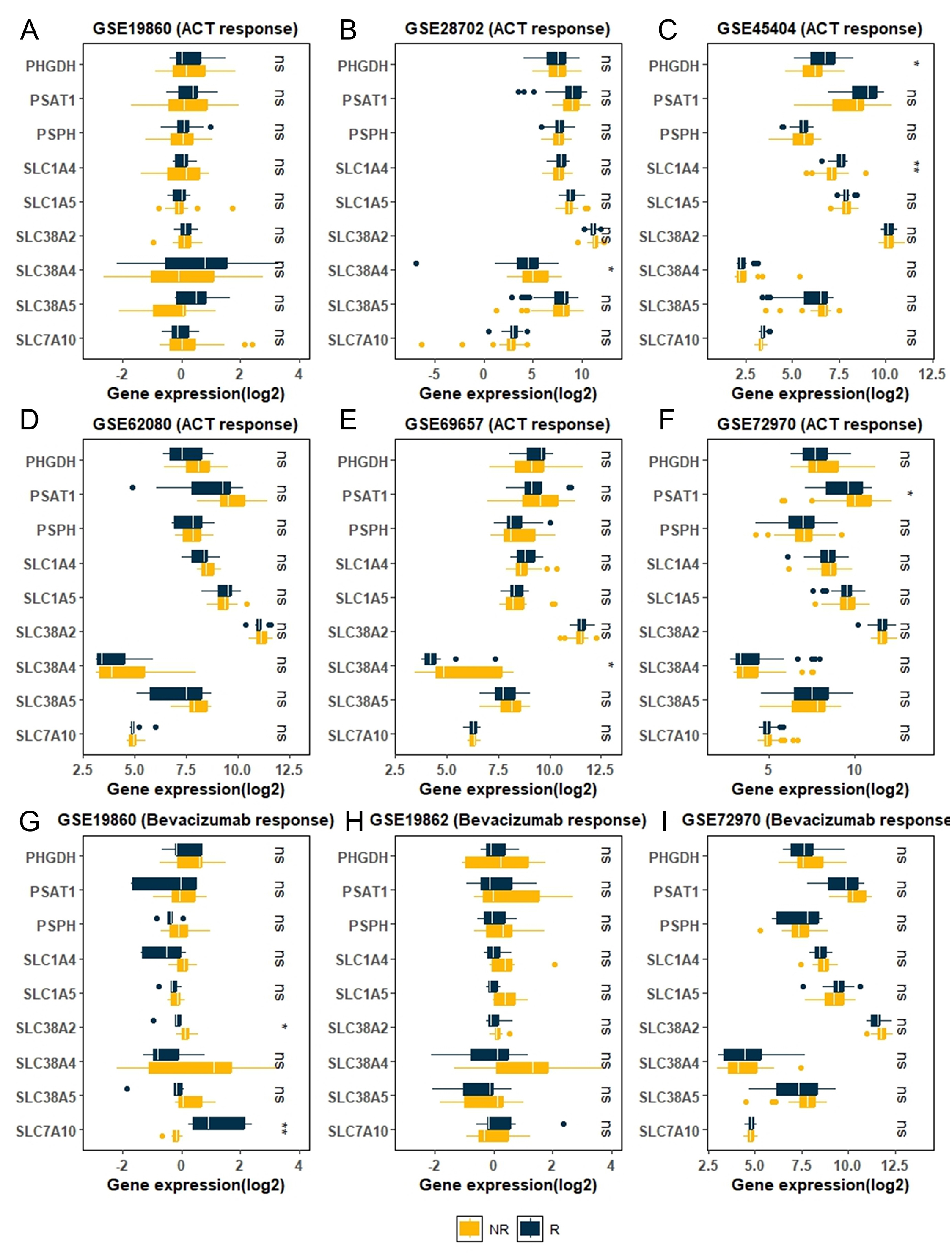
**

**Figure S9: Expression of SMGs in Fluorouracil-based ACT and Bevacizumab Benefits.**

(A-F) Box plots illustrating the distribution of SMG expression in responders and non-responders of fluorouracil-based ACT in GSE19860 (A), GSE28702 (B), GSE45404 (C), GSE62080 (D), GSE69657 (E), and GSE72970 (F) datasets.

(G-I) Box plots illustrating the distribution of SMG expression in responders and non-responders of bevacizumab in GSE19860 (G), GSE19862 (H), and GSE72970 (I) datasets.


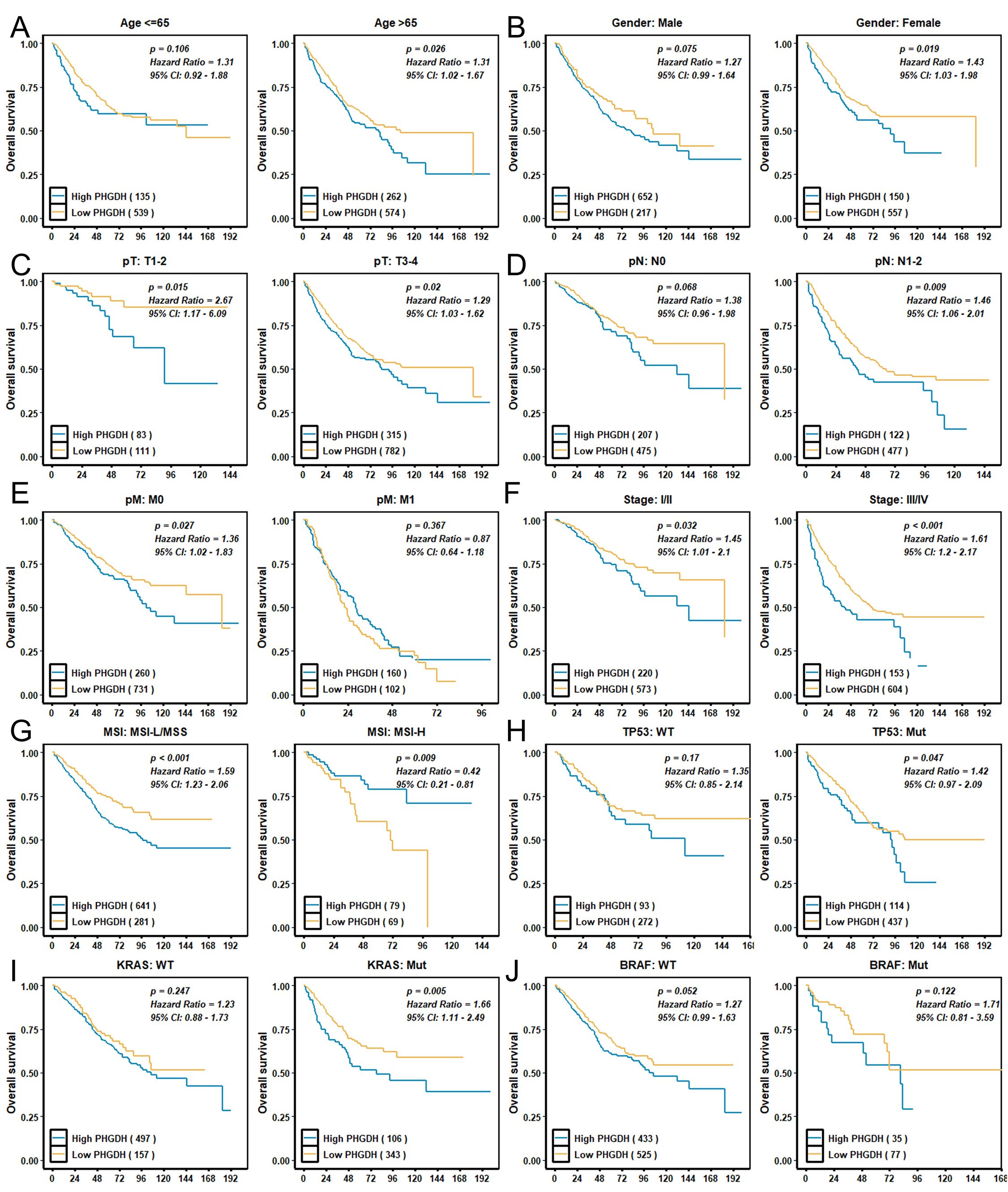


**Figure S10: Prognostic Value of SMGs in Colorectal Cancer Subgroups.**

(A-G) Kaplan-Meier survival analyses of PHGDH in CRC patients stratified by different clinical characteristics: age (A), gender (B), T stage (C), N stage (D), M stage (E), TNM stage (F), and MSI status (G).

(H-J) Kaplan-Meier survival analyses of PHGDH in CRC patients with TP53 (H), KRAS (I), and BRAF (J) mutations.


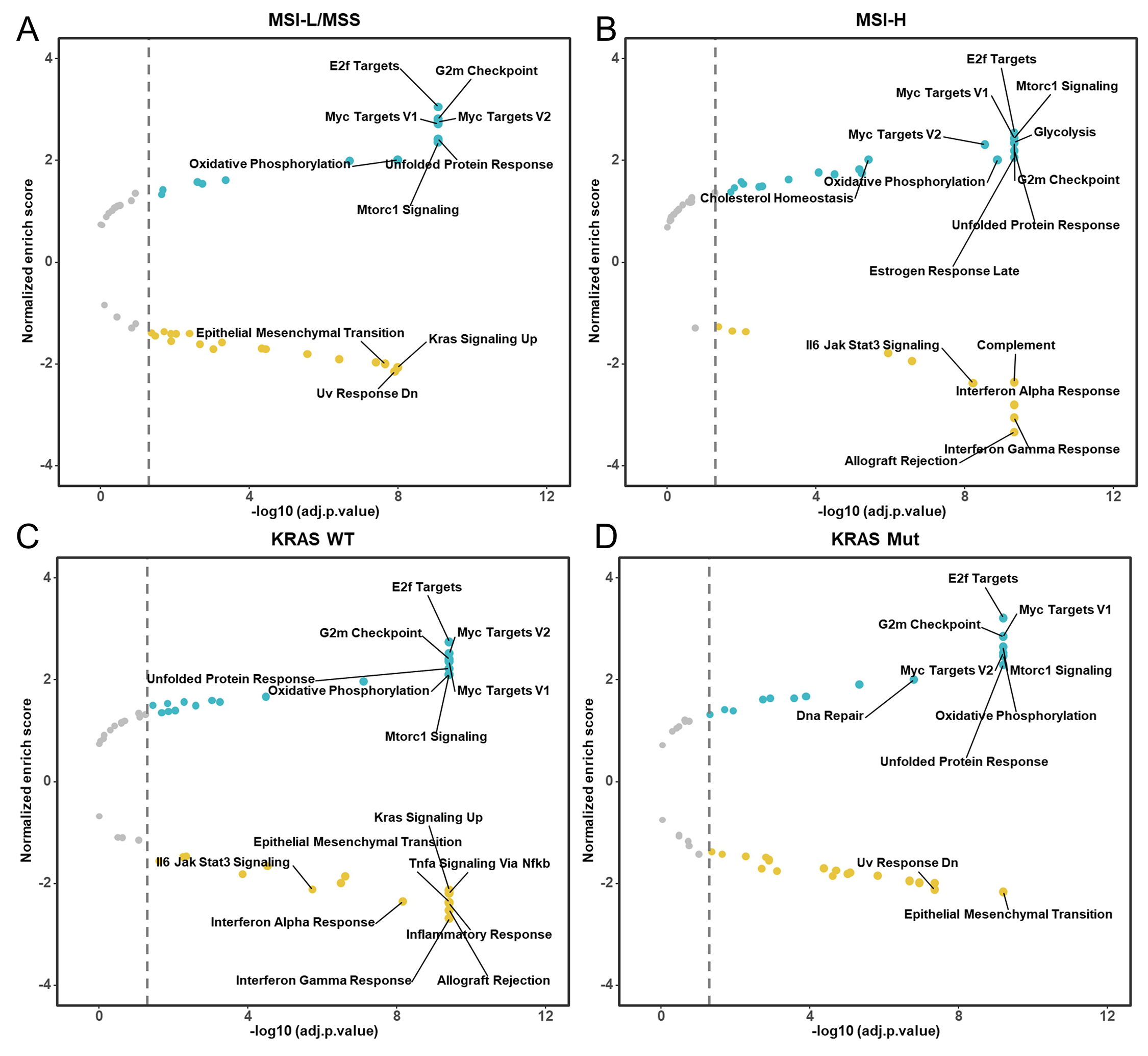


**Figure S11: GSEA Analysis Revealing Biological Functions of PHGDH in Different Colorectal Cancer Subgroups.**

(A-D) Volcano plots showing pathway enrichment differences between high and low PHGDH expression groups in CRC patients with different molecular characteristics: MSI-L/MSS (A), MSI-H (B), KRAS wild-type (C), and KRAS mutant (D).

**
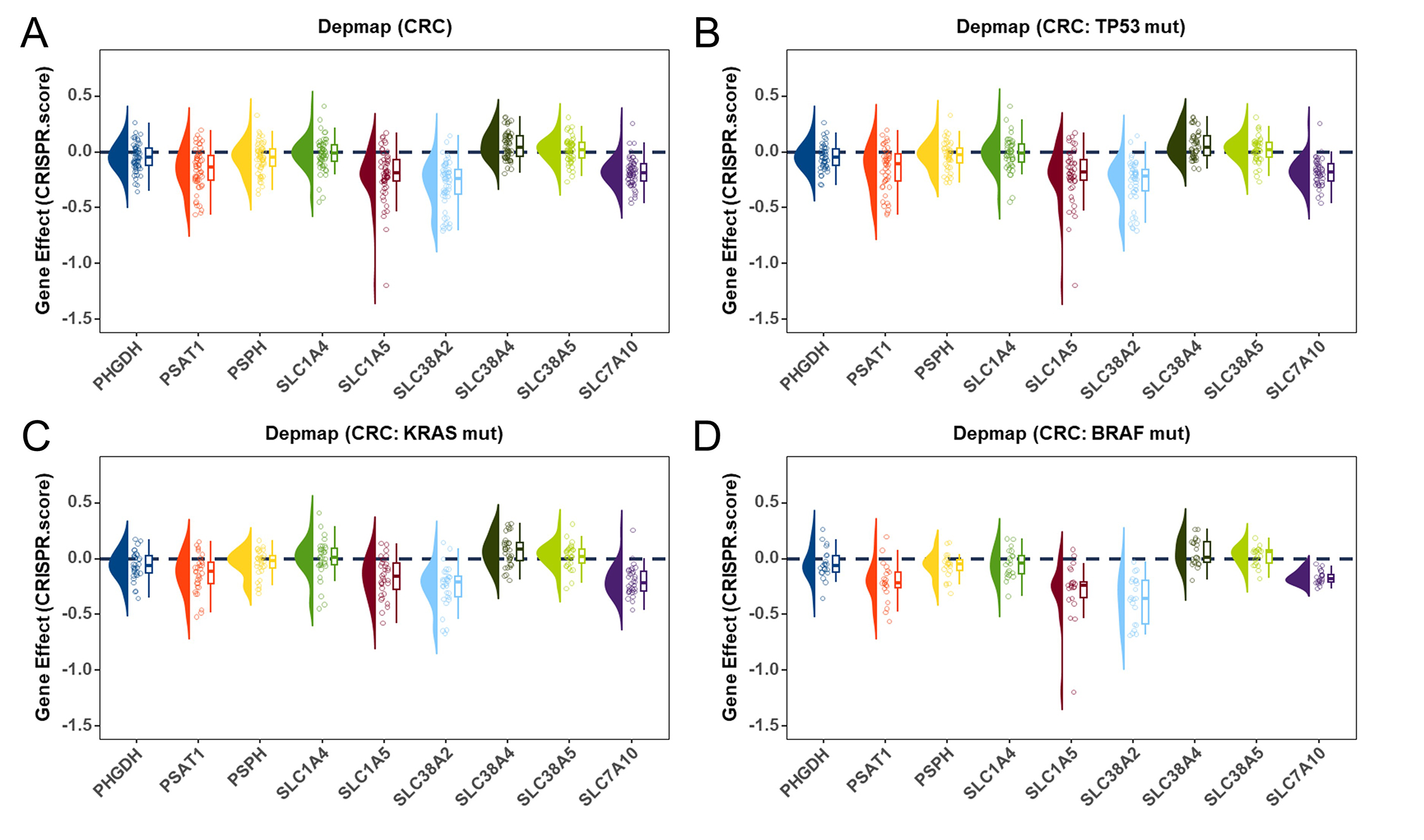
**

**Figure S12: Cell Viability Impact After Knockout of** **SMGs in the DepMap Database.**

1. D) Jitter plots showing CRISPR scores for SMGs across all CRC cell lines (A), TP53-mutant cells (B), KRAS-mutant cells (C), and BRAF-mutant cells (D).

**
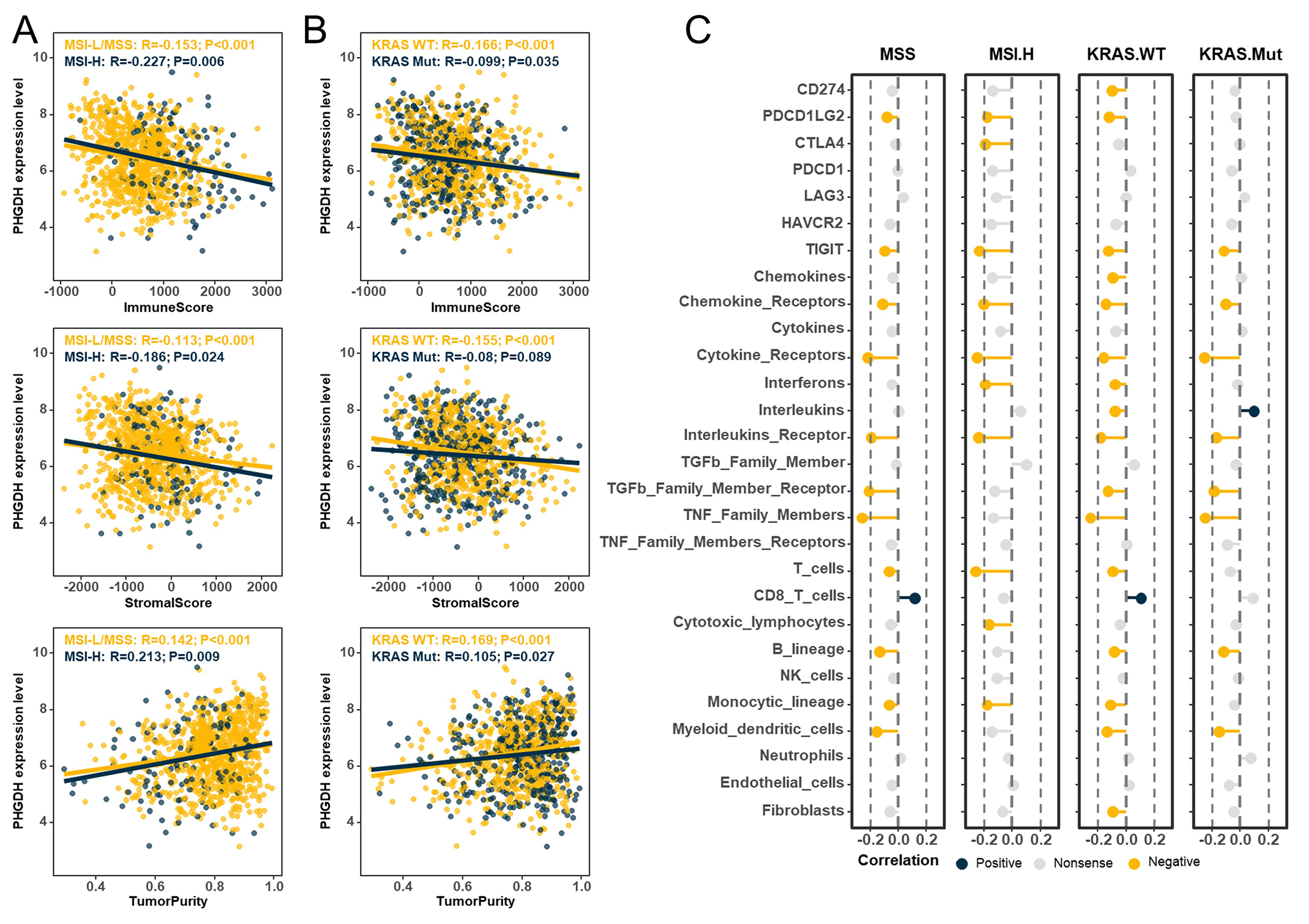
**

**Figure S13: Relationship Between PHGDH and TMEs in Pan-Cancer.**

(A, B) Correlation analyses of PHGDH with ImmuneScore, StromalScore, and tumor purity in CRC patients with different MSI status and KRAS mutation status.

(C) Correlations of PHGDH with immune checkpoints, immune cytokines score, and immune cells in CRC patients with different MSI status and KRAS mutation status.

**
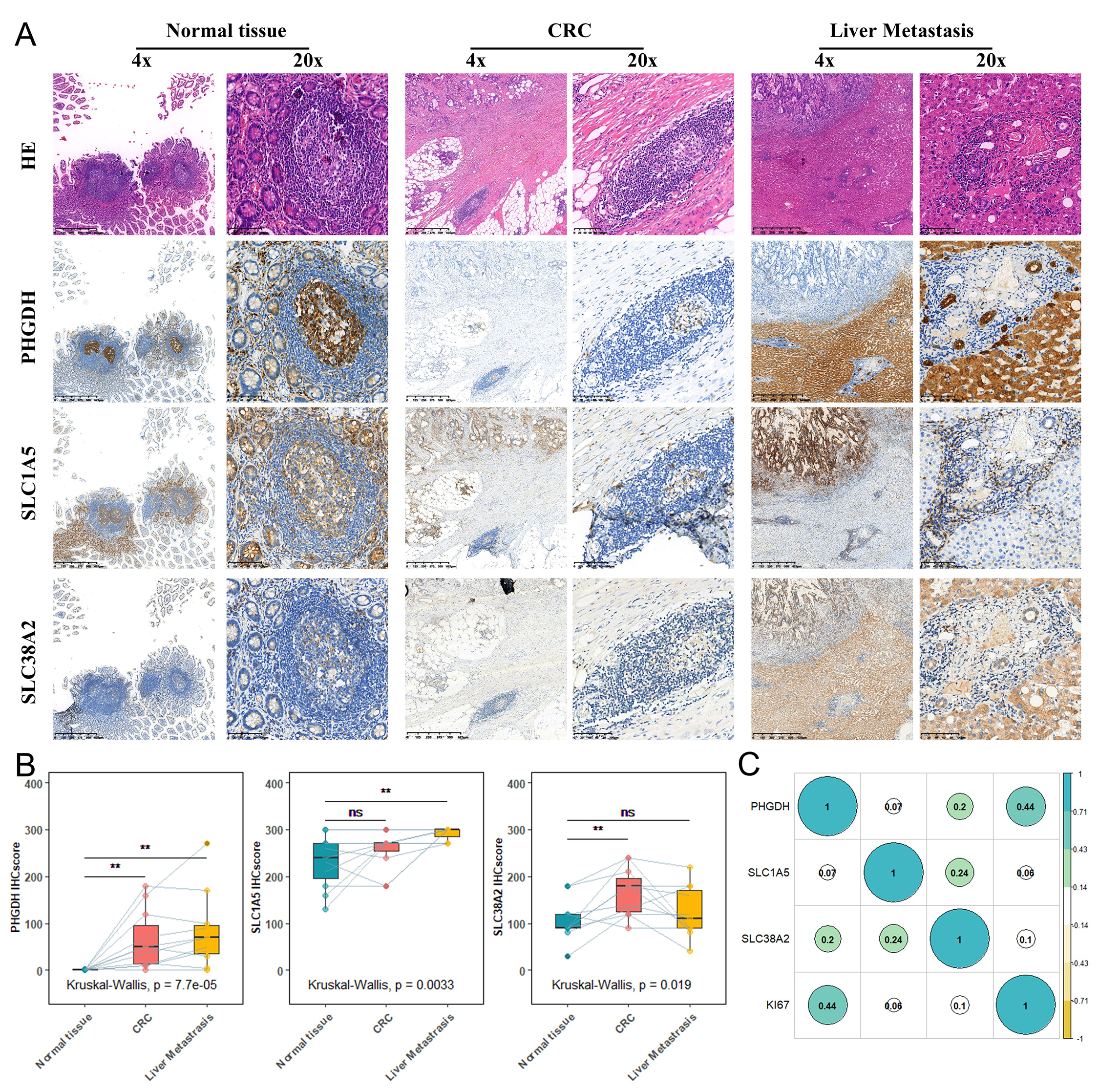
**

**Figure S14: Correlation of** **PHGDH, SLC1A5, and SLC38A2 Expression with Ki67 Proliferation Index in Real-World CRC Cohorts.**

1. Box plots showing the expression levels of PHGDH, SLC1A5, and SLC38A2 in cohort 1.
2. Correlation analyses between PHGDH, SLC1A5, and SLC38A2 and Ki67 proliferation index in CRC samples from cohort 2.


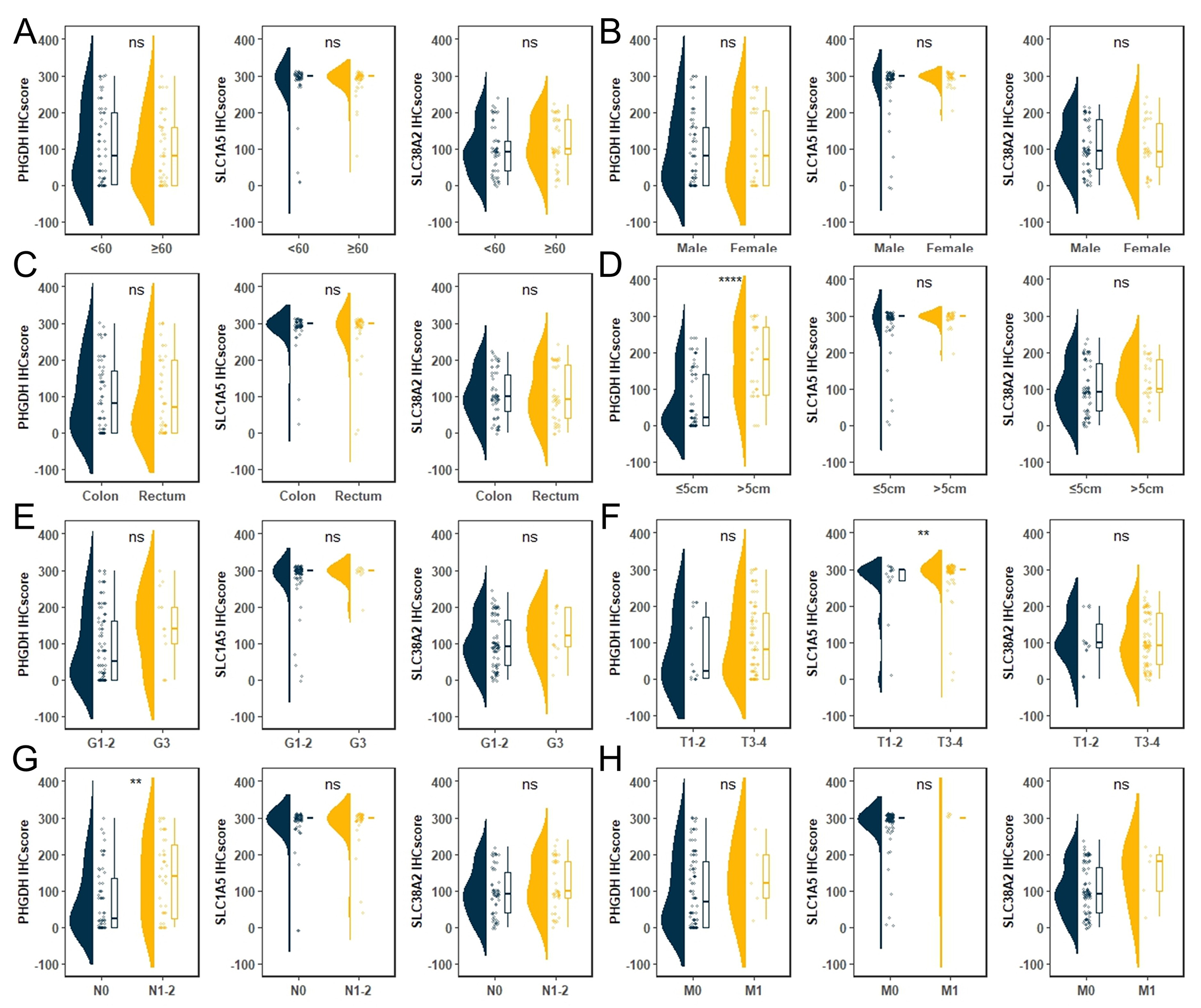


**Figure S15: Correlation Between PHGDH, SLC1A5, and SLC38A2 Expression and Clinical Features in Cohot2.**

(A-H) Expression levels of PHGDH, SLC1A5, and SLC38A2 (IHCscore) stratified by clinical characteristics of CRC patients in cohort 2, including age (A), gender (B), tumor location (C), tumor size (D), histological differentiation (E), T stage (F), N stage (G), and M stage (H).
